# Efficacy and safety of nonpharmacological strategies for the treatment of oligoasthenospermia: a systematic review and Bayesian network meta-analysis

**DOI:** 10.1186/s40001-022-00968-6

**Published:** 2023-01-04

**Authors:** Zhen Wang, Ziyang Zhou, Lijuan Zhang, Xujie Li, Miaoxiu Li, Yankun Pan, Tiyong Jiao, Xiaoyun Shi, Qing Liu, Congan Wang, Yongquan Wang

**Affiliations:** 1 Shandong University of Traditional Chinese Medicine, Jinan, China; 2 Neck, Shoulder, Waist and Leg Pain Hospital Affiliated to Shandong First Medical University, Jinan, China; 3 Affiliated Hospital of Shandong University of Traditional Chinese Medicine, Jinan, China

**Keywords:** Oligoasthenospermia, Nonpharmacological strategies, Network meta-analysis, Randomized controlled trials

## Abstract

**Background:**

Oligoasthenospermia (OAT) is the most common cause of male infertility, and the annual incidence of the disease continues to increase due to changing lifestyle habits, increased work pressure and increased environmental pollution. A variety of nonpharmacological therapies have been reported to be effective for treating OAT; however, there is a lack of direct evidence comparing these different nonpharmacological therapies. Therefore, the optimal strategy has yet to be identified.

**Objectives:**

A network meta-analysis was performed to evaluate the efficacy and safety of nonpharmacological treatments for OAT, thus providing an evidence-based medical reference for the clinical treatment of oligoasthenospermia.

**Methods:**

The Web of Science, Cochrane Library, Embase, PubMed, Weipu (VIP), Wan Fang Data, China National Knowledge Infrastructure (CNKI), and China Biomedical Literature (CBM) databases were searched from inception to April 2022 to identify randomized controlled trials (RCTs) that examined nonpharmacological treatments for oligozoospermia. Grey literature was also searched. Studies that met the quality criteria were analysed using Stata 16.0 and Review Manager 5.4 software.

**Results:**

A total of 4629 publications were initially retrieved; ultimately, 38 RCTs were analysed, including 8 nonpharmacological therapies and 3080 patients. Each intervention outperformed the sham intervention and no treatment approaches in terms of improved efficacy. In terms of improved total effective rate and sperm concentration, warming acupuncture may be the most effective treatment (SUCRA = 80.1% and 93.4%, respectively). Electroacupuncture perhaps resulted in the best improvement in sperm motility a% and a + b% (SUCRA = 96.6% and 82.0%, respectively). In terms of the incidence of adverse reactions, the three safest interventions probably were no treatment, warming acupuncture, and sham intervention (SUCRA = 88.0%, 68.8% and 62.9%, respectively). In terms of improving the reproductive hormones FSH, LH, and T, the best interventions perhaps were hyperbaric oxygen, 2 Hz TEAS, and electroacupuncture (SUCRA = 85.1%, 96.8% and 99.4%, respectively).

**Conclusions:**

Nonpharmacological treatments for oligoasthenospermia have good clinical efficacy. Warm acupuncture and electroacupuncture have better overall efficacy and safety. These treatment approaches can be recommended based on the actual situation. If a patient is complicated with varicoceles, they should be removed before symptomatic treatment. Due to the limitations of the quality of the included studies, the findings need to be further validated.

**Supplementary Information:**

The online version contains supplementary material available at 10.1186/s40001-022-00968-6.

## Background

Oligoasthenospermia (OAT) is a general term for oligospermia and asthenozoospermia and is an important cause of male infertility. Clinically, it is mainly characterized by decreased sperm concentration and sperm motility [1]. In recent years, under the influence of many harmful factors, such as environmental pollution, mental stress, and unhealthy lifestyles, the global annual prevalence of oligoasthenozoospermia among men has increased to 10–15%, leading to a heavy burden on individuals and the social health care system [2, 3]. Drugs are commonly used in clinical treatment for OAT. Western medicine mainly focuses on hormones and nutritional supplements (such as L-carnitine, vitamin C, E), while traditional Chinese medicine often uses drugs with the effect of invigorating the kidney and nourishing essence (such as Qilin Pill, Wuzi Yanzong Pill) for treatment. These drugs are still effective, but they easily reach the bottleneck of treatment, and there are many adverse reactions, such as gastrointestinal discomfort, arrhythmia and neurological lesions, which are often difficult for patients to tolerate [4, 5]. Therefore, it is necessary to explore other forms of alternative therapy with significant curative effects, stable effects and safety.

In recent years, the advantages of nondrug therapy for treating oligoasthenozoospermia have gradually emerged. It has the advantages of significant curative effects, rapid effects and few side effects and has been utilized by an increasing number of patients [6]. Several guidelines and consensuses [7, 8] list nondrug therapy as the recommended intervention for the clinical treatment of oligoasthenozoospermia, which can be mainly divided into surgical therapy (such as varicocelectomy and laser surgery), physical therapy (such as transcutaneous electrical acupoint stimulation, hyperbaric oxygen, and shock wave) and traditional Chinese medicine (acupuncture and massage). There are many types of nondrug therapies with different effects and advantages. It is not known which intervention measures have the best effect.

Based on the existing literature, we hypothesize that nondrug therapies can significantly improve the symptoms of oligoasthenospermia patients with high safety. However, the lack of evidence-based medicine has limited their wide application in clinical practice. Although multiple traditional meta-analyses [9–11] have proven that nondrug therapy has advantages in the treatment of oligoasthenozoospermia, most studies have compared two treatment methods (such as hyperbaric oxygen and drugs) rather than performing direct and indirect comparisons of multiple nondrug treatments. Therefore, the current study used the network meta-analysis method to compare the efficacy and safety of nondrug therapies commonly used in the treatment of oligoasthenospermia and to study the advantages of various methods in each outcome index to provide evidence-based medical support for the clinical treatment of oligoasthenospermia.

## Methods

This study followed the Preferred Reporting Items for Systematic Reviews and Meta-analysis (PRISMA-NMA) guidelines [12] and was registered with PROSPERO (Registration Number CRD42022314429).

### Inclusion criteria

#### Type of study

Randomized controlled trials (RCTs) published at home and abroad.

#### Research subjects

All studies met the diagnostic criteria for oligoasthenozoospermia [13–16], regardless of age or race.

#### Interventions

The experimental group was treated with nondrug therapy alone; the control group was treated with conventional medicine, sham intervention, or no treatment (e.g., electroacupuncture vs. manual acupuncture). The inclusion of intervention drugs in the control group refers to domestic and foreign guidelines or consensus [14, 17], including L-carnitine, vitamin C, E, zinc sulfate tablets, Qilin pills, and Wuziyanzong pills. Drugs need to be approved by the drug regulatory authorities for marketing. At least 3 articles are required for each nondrug therapy. A description of each intervention can be found in Additional file 1: Table S1.

#### Outcome indicator

Efficacy Indicator: total effective rate, referring to the efficacy standards formulated by the State Administration of Traditional Chinese Medicine and WHO [14, 15]. The total effective rate is calculated as follows: [(Cure + marked effect + effective) number of cases ÷ total number of cases] × 100%; sperm concentration; sperm motility a%; sperm motility a + b%. Safety indicators: adverse reaction. Laboratory Metrics: follicle-stimulating hormone, FSH; luteinizing hormone, LH; testosterone, T. All RCTs contained at least one of the outcome indicators to be eligible for inclusion in the NMA.

### Exclusion criteria

Inconsistent interventions; no mention of the outcome measures examined herein; no reference or homemade diagnostic criteria; incomplete or erroneous data; combined with serious complications.

### Data search and selection

The Cochrane Library, Web of Science, Embase, PubMed, VIP, CBM, CNKI and Wanfang databases were searched for relevant literature. We also searched grey literature and reviewed the reference lists of included studies and related systematic reviews. There were no restrictions regarding language, type of publication, date of publication or status of publication. The type of publication included original research, conference proceedings, letters to the editor, etc. The retrieval strategy used a combination of subject headings and free words, and the databases were searched from inception to April 1, 2022. An example search strategy from PubMed is provided in Additional file 1: Table S2. Two researchers (Li Miaoxiu and Zhang Lijuan) independently screened the literature based on the inclusion criteria. After extracting the data, they crosschecked each other’s results. Any disagreements were resolved by consulting a third party (Pan Yankun). Endnote software was used to check for duplicate publications. Then, the investigators screened the titles and abstracts of each study, and they excluded the literature that did not meet the inclusion criteria. Afterward, the investigators read the full texts of the remaining studies to decide whether to include it or not. If the literature was incomplete, the authors of the original study were contacted to obtain detailed data.

### Data extraction and bias assessment

Two reviewers (Jiao Tiyong and Shi Xiaoyun) independently extracted data from each trial using a standardized form. Any disagreements were resolved by consulting a third party (Liu Qing). The extracted data included the authors, publication time, sample size, disease duration and age, intervention measures, course of treatment, and outcome indicators. The risk of bias assessment was completed by 2 investigators (Li Xujie and Zhou Ziyang) using the risk of bias assessment tool (ROB2) in the Cochrane Reviewers Handbook [18]. The following 6 aspects were evaluated to determine the risk of bias: randomization process; deviations from intended interventions; missing outcome data; measurement of the outcome; selection of the reported result; and overall bias. Each studies was rated as “low risk”, “high risk” or “some concerns”.

### Quality of evidence

The GRADE (Grading of Recommendations Assessment, Development, and Evaluation) approach used to evaluate the quality of evidence for the primary outcomes and categorized as high, moderate, low, or very low. Two authors (Li Xujie and Zhang Lijuan) without conflicts of interest related to this study reviewed the synthesized evidence and downgraded its certainty based on study design, risk of bias, inconsistency, indirectness, and imprecision.

### Statistical analysis

All outcome indicators were analysed used random or fixed effects models based on the level of heterogeneity. The *P* value of the chi-square test and *I*^2^ index in the heterogeneity test were used to indicate the level of statistical heterogeneity. When the level of heterogeneity was low, the data were analysed with the fixed effects model (*P* ≥ 0.1 and *I*^2^ ≤ 50%); otherwise, the random effects model (*P* < 0.1 or *I*^2^ value > 50%) was used [19, 20]. The relative risk (RR) was used as the effect size for dichotomous variables, and the standardized mean difference (MD) was used as the effect size for continuous variables to calculate the 95% confidence interval (CI). Based on the Bayesian model, Stata 16.0 software was used for network meta-analysis. The data were preprocessed using the network group command, and the evidence network diagram of each indicator is drawn. The curative effect of the indicators was sorted to obtain the area under the curve (SUCRA), and the probability sorting was drawn as a graph. The dots in the evidence network diagram represent an intervention, and the larger the area is, the greater the number of patients with the intervention. The line connecting the two dots indicates a direct comparison between the two interventions, and the thickness of the line represents the number of included studies [21, 22]. The SUCRA is expressed as a percentage. The larger the percentage is, it means that the intervention has the highest probability and possibility of becoming the most preferred option, and a value of 0 indicates that the intervention may be completely ineffective [23, 24]. The transitivity assumption was assessed by comparing the characteristics of clinical and methodological variables and baseline information, such as patient age and trial design. When there is a closed loop, the node splitting method is used to check the inconsistency and transitivity. When the number of studies on the outcome indicator was > 10, a “comparison-adjusted” funnel plot was drawn to determine whether there was a possibility of a small sample effect. To test the robustness of the main findings, some factors that might have a potential to influence the level of precision of the main outcome were removed and sensitivity analysis was performed.The quality of the literature was evaluated by Review Manager 5.4 software.

## Results

### Literature search and study characteristics

**Fig. 1:**
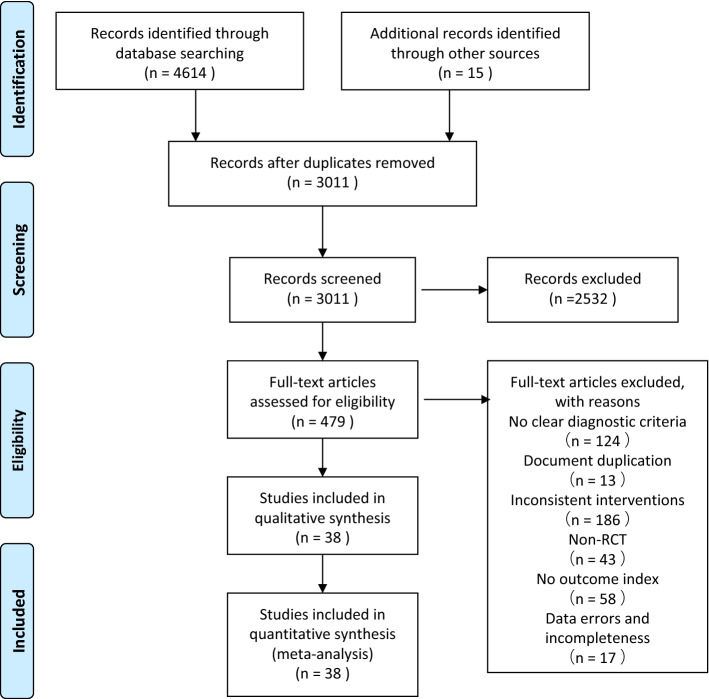
Literature search process

The search yielded 4629 studies, of which 38 [25–62] were ultimately included. Three studies [33, 34, 54] were three-arm trials, four studies [35–38] were four-arm trials, and the remaining studies were two-arm trials. The screening flow chart is shown in Fig. 1. A total of 3080 patients were included, all of whom met the diagnostic criteria for oligoasthenozoospermia; there were 1462 patients in the experimental group and 1618 patients in the control group. There were 8 nondrug therapies, including electroacupuncture, 2 Hz TEAS, 100 Hz TEAS, warming acupuncture, moxibustion, manual acupuncture, varicocelectomy, and hyperbaric oxygen. Most of the patients in these studies were 25–35 years. All included studies were rigorous RCTs. Therefore, the transitivity assumption was met based on baseline characteristics. The basic characteristics of the included studies are shown in Table 1.

**Table 1:** Basic characteristics of the included studies

Included studies	Mean age/years		Sample size		Mean disease duration/year		Interventions		Duration/month	Outcome measures
	T	C	T	C	T	C	T	C		
Zhang et al. [23]	21 ~ 52	20 ~ 46	80	80	1 ~ 4	1 ~ 3.5	EA	CM	3 months	①
Pan [24]	32.24 ± 5.02	33.37 ± 4.29	31	30	3.16 ± 1.53	2.93 ± 1.46	EA	CM	3 months	②④⑤
Wang et al. [25]	26.3 ± 4.2	26.5 ± 3.9	71	82	3.1 ± 0.6	2.9 ± 0.5	EA	CM	3 months	①②③⑤
Zhou et al. [26]	32.1 ± 5.8	33.9 ± 6.5	32	33	4.2 ± 2.1	3.8 ± 2.0	EA	CM	3 months	①②④⑤
Cao et al. [27]	32.40 ± 4.83	32.54 ± 4.16	30	30	3.54 ± 1.38	3.62 ± 1.26	EA	CM	2 months	①②③
Li [28]	33.23 ± 3.37	31.90 ± 2.58	48	47	21.87 ± 6.71 months	18.10 ± 6.39mon	EA	MA	3 months	①②④⑤⑥⑦⑧
Li et al. [29]	32.14 ± 3.37	29.95 ± 3.32	40	40	4.75 ± 2.35	5.05 ± 2.63	2 Hz TEAS	CM	2 months	①②④⑤⑥⑦⑧
Fang et al. [30]	32 ± 3	32 ± 3	35	35	4.6 ± 1.8	4.9 ± 1.4	2 Hz TEAS	CM	3 months	①②③④⑤
Chi and Ge [31]	31.21 ± 2.32	29.61 ± 2.09	31	30	2.33 ± 0.99	2.61 ± 1.52	2 Hz TEAS	MA	3 months	④⑥⑦⑧
		33.03 ± 1.72		30		2.43 ± 1.24		SI		
Jin et al. [32]	32.68 ± 0.95	32.00 ± 1.10	25	16	–	–	2 Hz TEAS	100 Hz TEAS	2 months	①②③④
		30.33 ± 0.91		15		–		NT		
Dong [33]	30.75 ± 4.30	32.24 ± 3.56	20	20	–	–	2 Hz TEAS	100 Hz TEAS	1 month	①③④⑤
		28.64 ± 3.37		21		–		SI		
		30.50 ± 5.13		22		–		NT		
Zhang et al. [34]	23 ~ 40	23 ~ 40	20	20	–	–	2 Hz TEAS	100 Hz TEAS	1 month	①③④
		23 ~ 40		21		–		SI		
		23 ~ 40		22		–		NT		
Sha [35]	31.89 ± 3.32	32.24 ± 3.47	28	26	3.5 ± 1.5	4.5 ± 1.6	2 Hz TEAS	100 Hz TEAS	2 months	①②④⑤⑥⑦⑧
		31.43 ± 3.38		21		3.6 ± 1.4		SI		
		31.67 ± 5.06		30		4.5 ± 1.6		NT		
Yu et al. [36]	31.45 ± 0.84	30.94 ± 1.07	31	31	4.65 ± 0.26	4.85 ± 0.36	2 Hz TEAS	100 Hz TEAS	2 months	②④
		29.52 ± 0.81		29		5.02 ± 0.41		SI		
		31.70 ± 0.98		30		5.37 ± 0.32		NT		
Liu et al. [37]	25 ~ 35	25 ~ 35	40	40	1 ~ 6	1 ~ 6	MB	CM	3 months	①②④
Han et al. [38]	25 ~ 49	25 ~ 49	32	32	–	–	MB	CM	3 months	①
Yang and Qin [39]	32.19 ± 5.82	31.58 ± 5.87	31	31	–	–	MB	CM	3 months	①
Jia et al. [40]	44.35 ± 4.36	45.25 ± 3.45	20	20	22.65 ± 2.87 months	23.00 ± 3.28 months	MB	CM	3 months	②⑤
Zhang [41]	29 ± 4.6	30 ± 5.7	30	30	1.8 ± 0.9	1.9 ± 0.8	MA	NT	3 months	②③④⑥⑦⑧
Shi [42]	26.3 ± 2.6	26.6 ± 2.8	31	33	3.2 ± 0.5	3.4 ± 0.6	MA	CM	6 months	①②③⑤
Yang and Zhang [43]	25 ~ 38	25 ~ 38	50	50	1 ~ 6	1 ~ 6	MA	SI	10 weeks	①⑤
Jiang et al. [44]	30.3 ± 5.6	29.4 ± 5.1	26	26	12.8 ± 4.4 months	12.1 ± 4.7	MA	CM	2 months	②④
Sun et al. [45]	32 ± 3	31 ± 3	42	40	6.4 ± 0.5	6.3 ± 0.3	MA	SI	10 weeks	①②④
Li [46]	31.5 ± 3.3	30.5 ± 2.8	42	40	6.4 ± 0.5	6.3 ± 0.3	MA	SI	10 weeks	①②④⑥⑦⑧
Ding et al. [47]	28.6 ± 6.9	25.5 ± 8.1	25	26	20.6 ± 13.2 months	17.6 ± 12.7 months	MA	CM	1 month	③④⑤
Siterman et al. [48]	39 ± 7	39 ± 7	20	20	9 ± 4	9 ± 4	MA	NT	5 weeks	②
Dieterle et al. [49]	–	–	24	28	–	–	MA	SI	6 weeks	②③
Su [50]	34.9 ± 0.58	35.0 ± 0.61	30	30	–	–	MA	CM	3 months	①②④⑥⑦⑧
Wang et al. [51]	26.38 ± 3.54	26.16 ± 3.16	37	38	35.62 ± 5.61 months	34.84 ± 4.65 months	MA	CM	3 months	①②③④⑤
Sun et al. [52]	28.25 ± 2.28	27.97 ± 2.30	33	34	2.45 ± 0.39	2.41 ± 0.38	WA	MA	2 months	①②③④⑥⑦⑧
		28.15 ± 2.53		33		2.52 ± 0.33		CM		
Jia et al. [53]	17 ~ 35	17 ~ 35	20	10	3 months ~ 4 years	3 months ~ 4 years	VCL	NT	4 months	②④⑤⑥⑦⑧
Gu et al. [54]	28 ~ 45	28 ~ 45	33	33	1 ~ 5	1 ~ 5	VCL	CM	3 months	①②④⑤
Li et al. [55]	17 ~ 35	17 ~ 35	19	16	–	–	VCL	NT	3 months	②④⑥⑦⑧
Baazeem et al. [56]	34.9 ± 5.5	36.2 ± 5.5	233	127	2.85 ± 2.42	3.05 ± 2.14	VCL	NT	3 months	②④
Chen and Zheng [57]	34.46 ± 3.02	34.55 ± 2.98	40	40	2.65 ± 1.07	2.70 ± 0.95	HBO	CM	1 month	①②④⑤
Chen et al. [58]	33.26 ± 5.59	33.08 ± 5.41	30	30	3.13 ± 0.62	3.08 ± 0.57	HBO	CM	1 month	②③④⑥⑦⑧
Zhao et al. [59]	27.2 ± 5.0	26.9 ± 3.6	28	24	–	–	HBO	CM	3 months	①
Zhang et al. [60]	23 ~ 38	24 ~ 37	24	26	–	–	HBO	CM	3 months	①

*T* Test group, *C* Control group, *–* It was not mentioned, *EA* Electroacupuncture, *TEAS* Transcutaneous electrical acupoint stimulation, *MB* Moxibustion, *MA* Manual acupuncture, *WA* Warming acupuncture, *VCL* Varicocelectomy, *HBO* Hyperbaric oxygen, *CM* conventional medicine, *SI* Sham intervention, *NT* No treatment, ①Total effective rate; ②Sperm concentration; ③Sperm motility a%; ④Sperm motility a + b%; ⑤Adverse reaction; ⑥FLH. Follicle-stimulating hormone; ⑦ LH. Luteinizing hormone; ⑧ T. Testosterone. The course of treatment of VCL is the postoperative follow-up time, and the control group is the duration of treatment

### Bias and GRADE assessment

**Fig. 2:**
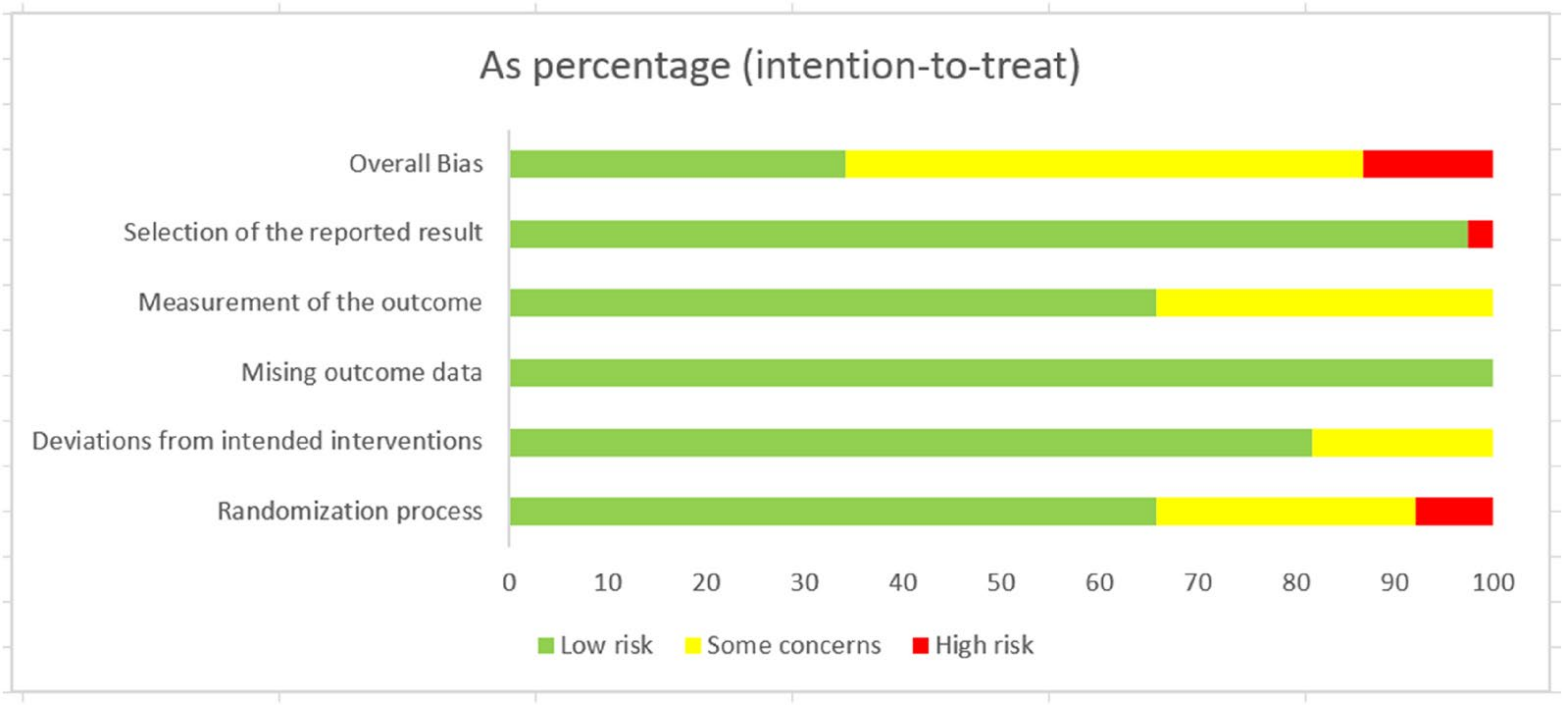
Literature bias evaluation results

Of the 38 included RCTs, five studies [38, 50, 51, 57, 58] were published in English, and the remainder were published in Chinese. The studies had comparable general information for the control and experimental groups. In the evaluation of the risk of bias regarding the randomization process, only 3 studies [27, 44, 60] were evaluated as “high risk”, 10 articles [25, 28, 29, 36, 39, 40, 49, 53, 58, 62] were evaluated as having “some concerns”, and 25 articles were evaluated as “low risk”. In the evaluation of the risk of bias regarding deviations from intended interventions, 7 studies [27, 29, 32, 39, 40, 46, 59] were rated as having “some concerns”, and the remaining 31 articles were rated as “low risk”. In the evaluation of the risk of bias regarding missing outcome data, all 38 studies were rated “low risk”. In the evaluation of the risk of bias regarding measurement of the outcome, 13 studies [29, 32, 33, 35, 36, 39, 43–45, 47, 48, 52, 62] were rated as having “some concerns”, and the remaining 25 studies were rated as “low risk”. In the evaluation of the risk of bias regarding selection bias, only 1 study [54] was evaluated as “high risk”, and the remaining 37 studies were rated as “low risk”. According to the ROB2.0 risk of bias assessment tool, 15 studies [25, 28, 29, 32, 33, 35, 36, 40, 41, 43, 47–49, 52, 53, 55, 58, 59, 62] were rated as having an overall “low risk”, 18 articles were rated as having a risk of bias for a “some concerns”, and 5 articles [27, 39, 44, 50, 54] were rated as having an overall “high risk”. The results are shown in Fig. 2, and the risk of bias summary is shown in Additional file 1: Figure S1. GRADE results is shown in Additional file 1: Figure S9. In terms of importance, other outcome indicators showed important in addition to the total effective rate, sperm concentration, sperm motility a%. Moreover, the quality of evidence was very low or low because of the poor methodological quality.

### Network meta-analysis

#### Evidence network diagram, inconsistency and transitivity test

##### Total effective rate

**Fig. 3:**
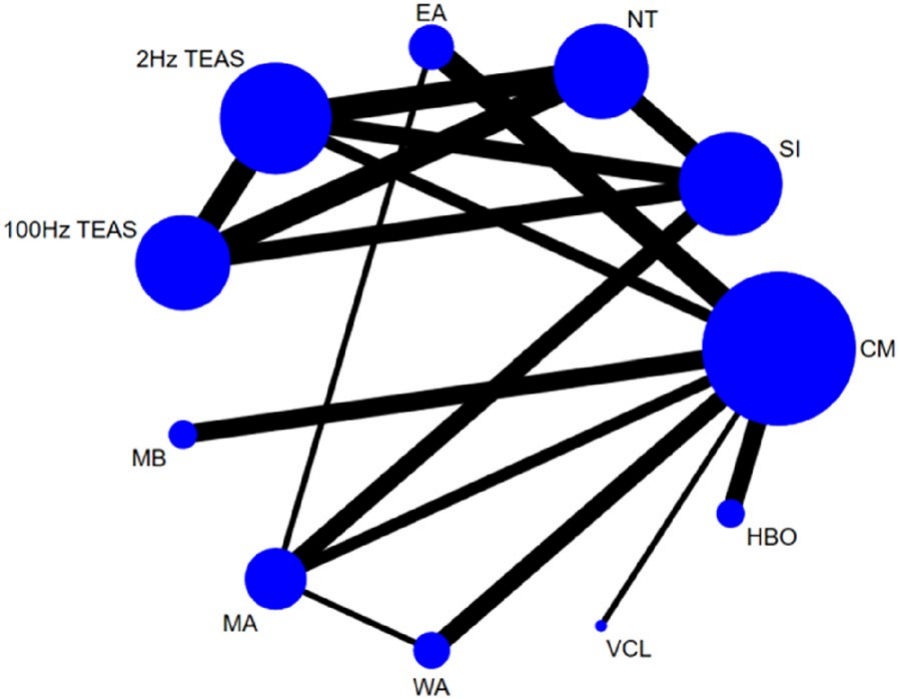
Network diagram of the total effective rate

Twenty-five studies [25, 27–32, 34–37, 39–41, 44, 45, 47, 48, 52–54, 56, 59, 61, 62] reported the total effective rate, involving a total of 11 interventions. Thus, 55 two-by-two comparisons were formed, and the evidence network was generally centred on CM, thereby forming six closed loops (see Fig. 3). Due to the low level of heterogeneity (*P* = 0.115, *I*^2^ = 23.5%), we used a fixed effects model. The node-splitting method test showed good consistency and transitivity, with no heterogeneity emerging between studies (*P* > 0.05).

##### Sperm concentration

**Fig. 4:**
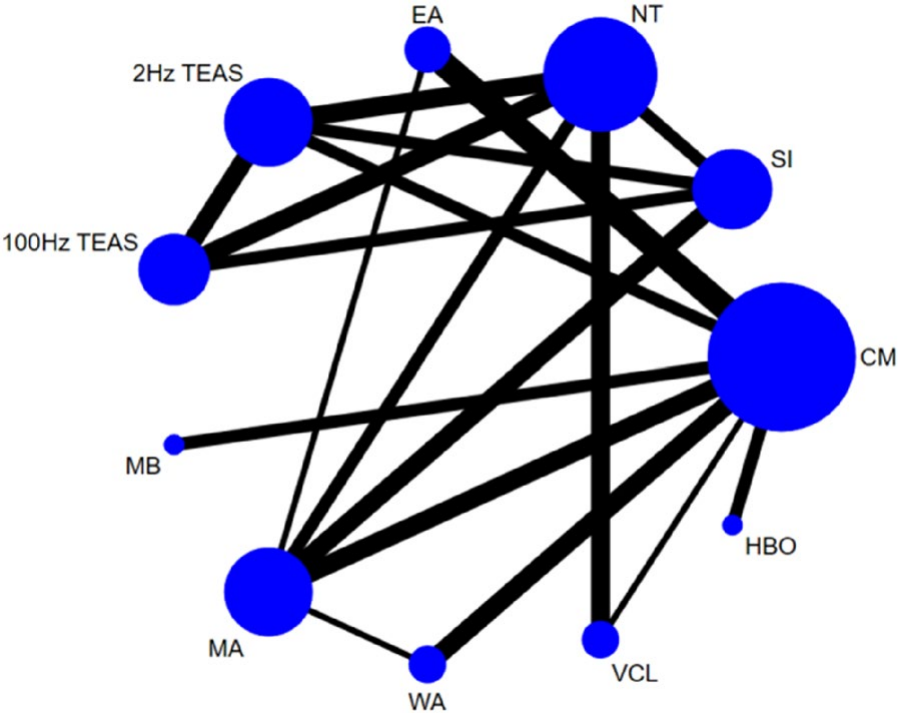
Network diagram of sperm concentration

Twenty-eight studies [26–32, 34, 37–39, 42–44, 46–48, 50–60] reported on sperm concentration, involving 11 interventions. Thus, 55 two-by-two comparisons were formed, and the evidence network was generally centred on CM, thereby forming eight closed loops (see Fig. 4). Due the high amount of heterogeneity (*P* < 0.00001, *I*^2^ = 88.4%), we used a random effects model. Node-splitting tests showed good transitivity and agreement, with no heterogeneity between studies (*P* > 0.05).

##### Sperm motility a%

**Fig. 5:**
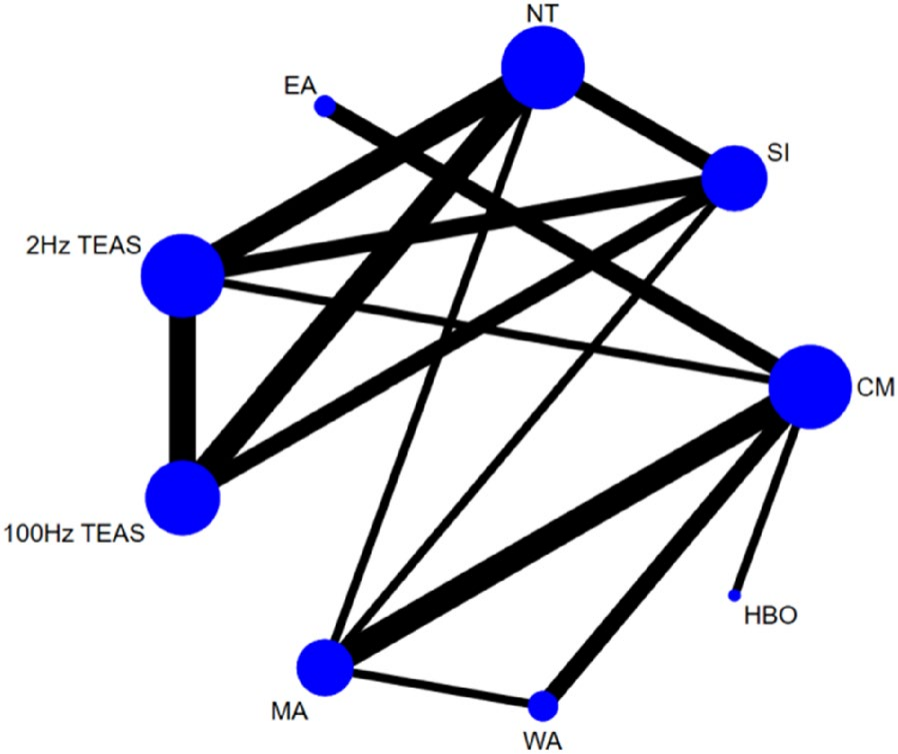
Network diagram of sperm motility a%

Thirteen studies [27, 29, 32, 34–36, 43, 44, 49, 51, 53, 54, 60] reported sperm motility a%, involving nine interventions. Thus, 36 two-by-two comparisons were formed, leading to six closed loops (see Fig. 5). Due to the high amount of heterogeneity (*P* < 0.00001, *I*^2^ = 84.9%), we used a random effects model. Node-split tests showed good agreement and transitivity, with no heterogeneity emerging between studies (*P* > 0.05).

##### Sperm motility a + b%

**Fig. 6:**
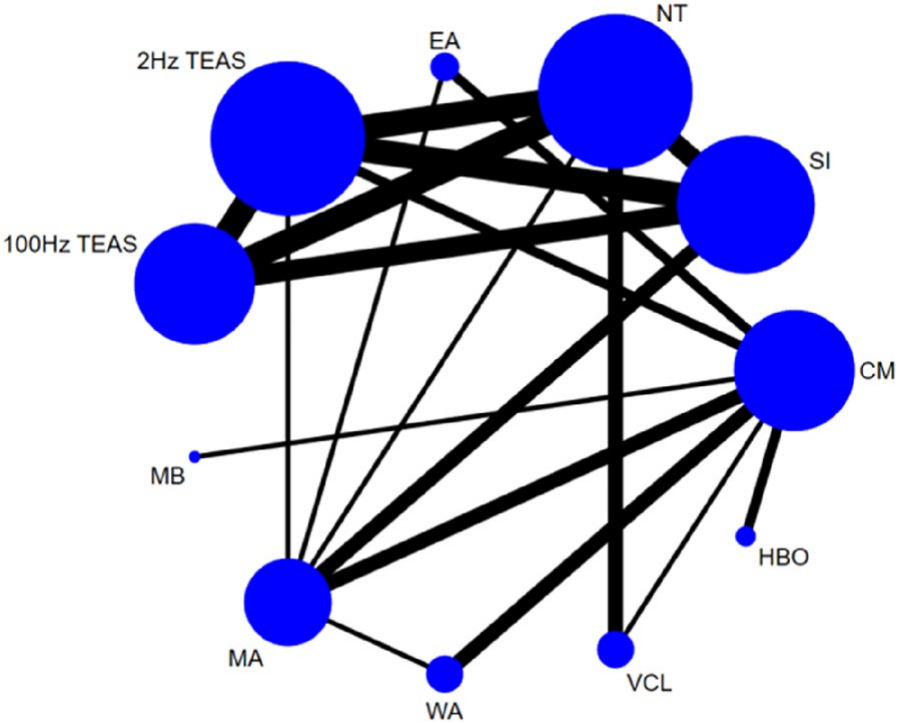
Network diagram of sperm motility a + b%

Twenty-six studies [26, 28, 30–39, 43, 46–49, 52–60] reported sperm motility a + b%, involving 11 interventions. Thus, 55 two-by-two comparisons were formed, with an overall network of evidence centred on no treatment, thereby forming nine closed loops (see Fig. 6). Due to the high amount of heterogeneity (*P* < 0.00001, *I*^2^ = 92.7%), we used a random effects model. Node-split tests showed good transitivity and consistency, with no heterogeneity emerging between studies (*P* > 0.05).

##### Adverse reaction

**Fig. 7:**
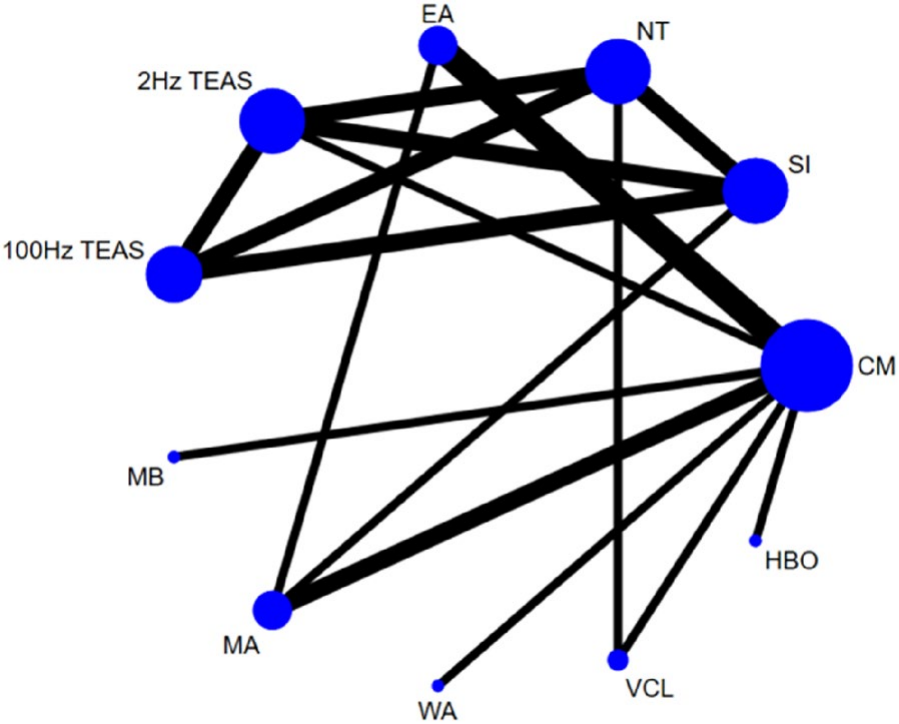
Network diagram of adverse reactions

Of the 38 articles, 15 reported the occurrence of adverse reactions. Among them, 8 [26–28, 35, 37, 42, 44, 45] had no adverse reaction; 7 studies [30, 32, 49, 53, 55, 56, 59] reported minor adverse reactions and no serious adverse reactions. Due to the limited number of included literatures and the broad definition, adverse reactions could not be specifically subdivided, and only the total number of adverse reactions from interventions was analyzed. Specific adverse reactions are detailed in the Additional file 1: Table S23. Fifteen studies [26–28, 30–32, 35, 37, 42, 44, 45, 49, 53, 55, 56, 59] reported adverse reactions, involving 11 interventions. Thus, 55 two-by-two comparisons were formed, and the evidence network was generally centred on CM, thereby forming nine closed loops (see Fig. 7). Due to the low amount of heterogeneity (*P* = 0.923, *I*^2^ = 0.0%), we used a fixed effects model. Node-splitting tests showed good transitivity and agreement, with no heterogeneity between studies (*P* > 0.05).

##### FSH, LH, T

**Fig. 8:**
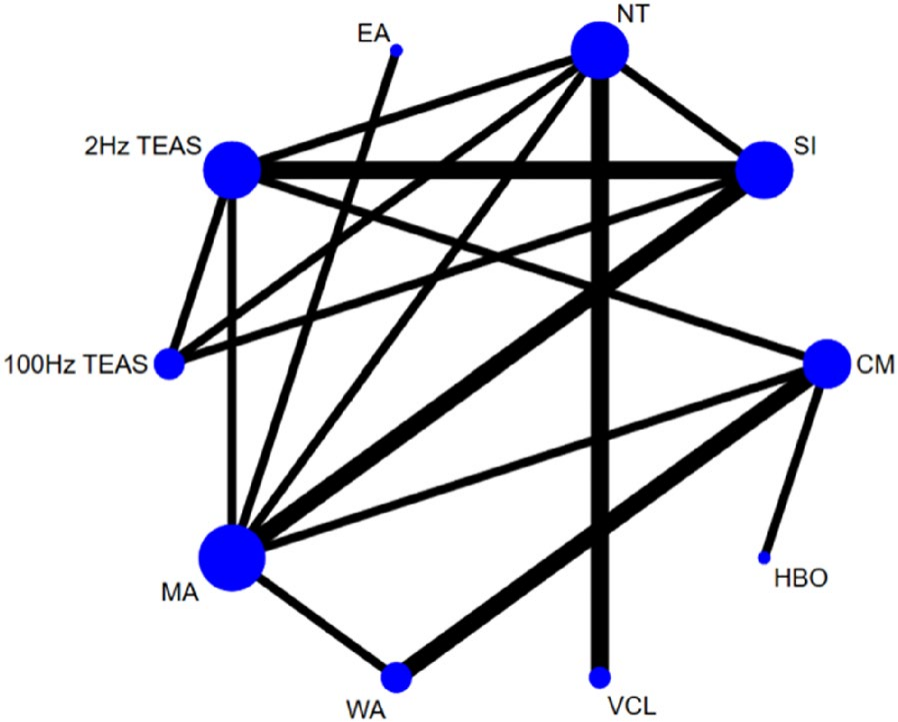
Network diagram of FSH, LH, and T

As the results of the included studies that examined these 3 indicators were consistent, their evidence network plots were the same and thus pooled for analysis. Eleven studies [30, 31, 33, 37, 43, 48, 52, 54, 55, 57, 60] reported on FSH, LH and T, involving a total of 10 interventions. Thus, 45 two-by-two comparisons were formed, and the evidence network was generally centred on manual acupuncture as the centre, thereby forming seven closed loops (see Fig. 8). The three indicators have high heterogeneity; the *I*^2^ values were 87.1%, 90.7% and 82.9%, respectively, and the *P* values were less than 0.00001. Therefore, the random effects model was used for these three indicators. The results of the node-splitting method test showed good consistency and transitivity, with no heterogeneity emerging between studies (*P* > 0.05).

The results of the node-splitting tests are shown in Additional file 1: Tables S3, S4, S4, S5, S6, S7, S8, S9, S10.

#### Results of the network meta-analysis

##### Total effective rate

WA was better than CM [RR = 1.15, 95% CI (1.01, 1.31)] and MA [RR = 1.28, 95% CI (1.05, 1.56)] in terms of improving the total effective rate. Compared with NT, WA, EA, MB, 2 Hz TEAS, HBO, 100 Hz TEAS, CM, VCL, MA and SI had better intervention effects. EA was better than MA. WA, EA, MB, 2 Hz TEAS, HBO, 100 Hz TEAS, CM and MA were better than SI. All of the abovementioned differences were statistically significant (*P* < 0.05), as shown in Table 2.

**Table 2:**
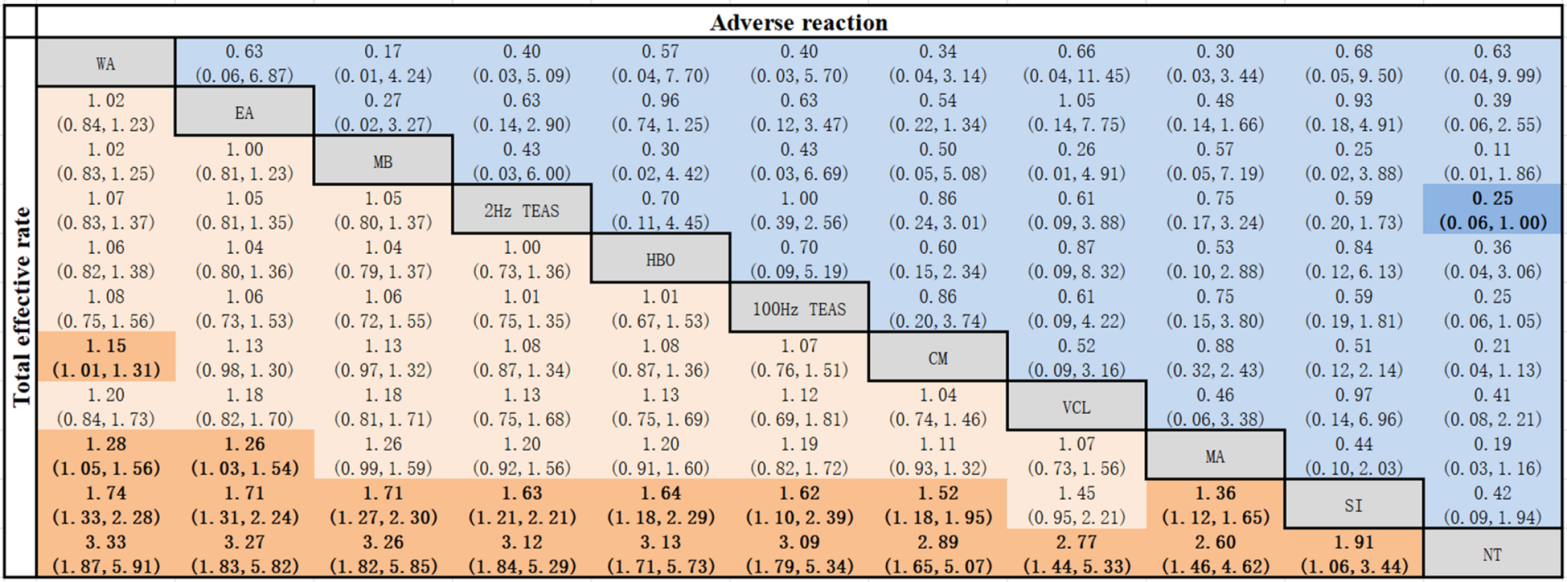
Network meta-analysis of the total effective rate and adverse reaction rate [RR (95% CI)]

Bold values are statistically significant; Tables 2, 3, 4, 5 are the same.

##### Adverse reaction

2 Hz TEAS was superior to NT in improving the ate of adverse reactions [RR = 0.25, 95% CI (0.06, 1.00)], with a statistically significant difference (*P* < 0.05). There were no statistically significant differences (*P* > 0.05) when comparing the remaining interventions (see Table 2).

##### Sperm concentration

Compared to 100 Hz TEAS, WA [MD = 6.67, 95% CI (2.44, 10.90)], MB [MD = 5.72, 95% CI (1.12, 10.32)], MA [MD = 4.13, 95% CI (0.63, 7.62)], EA [MD = 4.11, 95% CI (0.21 8.00)], CM [MD = 4.05, 95% CI (0.70, 7.40)], 2 Hz TEAS [MD = 3.56, 95% CI (0.73, 6.39)] were better at elevating sperm concentration. WA, MB, HBO, MA, EA, CM, 2 Hz TEAS were better than VCL, SI, and NT. 100 Hz TEAS was better than SI and NT. VCL was a better intervention than NT. All of the abovementioned differences were statistically significant (*P* < 0.05), as shown in Table 3.

**Table 3:**
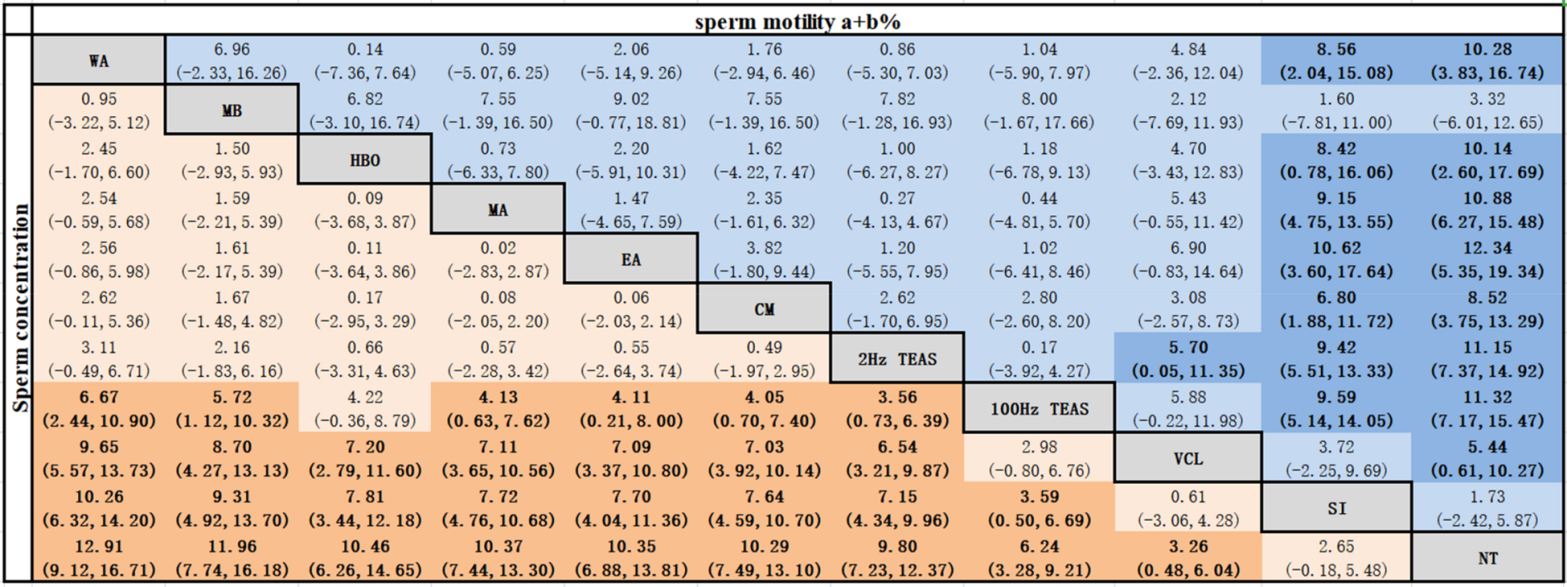
Network meta-analysis of sperm concentration and sperm motility a + b% [MD (95% CI)]

Bold values are statistically significant; Tables 2, 3, 4, 5 are the same.

##### Sperm viability a + b%

VCL [MD = 5.44, 95% CI (0.61, 10.27)] was more effective are enhancing sperm viability a + b% than NT. EA, 100 Hz TEAS, 2 Hz TEAS, MA, WA, HBO and CM were better than SI and NT. 2 Hz TEAS was a better intervention than VCL. All of the abovementioned differences were statistically significant (*P* < 0.05), as shown in Table 3.

##### Sperm viability a%

Compared to SI, EA [MD = 7.68, 95% CI (3.71, 11.66)], 100 Hz TEAS [MD = 5.18, 95% CI (1.94, 8.41)], HBO [MD = 4.55, 95% CI (0.13, 8.97)], and CM [MD = 4.50, 95% CI (1.17, 7.83)] were more effective at enhancing sperm viability grade a. EA was better than CM, MA, and 2 Hz TEAS. WA was better than MA. 100 Hz TEAS was better than 2 Hz TEAS. Compared with NT, EA, WA, 100 Hz TEAS, HBO, CM, MA, and 2 Hz TEAS were more effective interventions. All of the abovementioned differences were statistically significant (*P* < 0.05), as shown in Table 4.

##### T

Compared to SI, MA [MD = 1.84, 95% CI (0.39, 3.28)] was better at elevating T. EA was better than WA, MA, CM, HBO, VCL, 100 Hz TEAS, 2 Hz TEAS, SI and NT. All of the abovementioned differences were statistically significant (*P* < 0.05), as shown in Table 4.

**Table 4:**
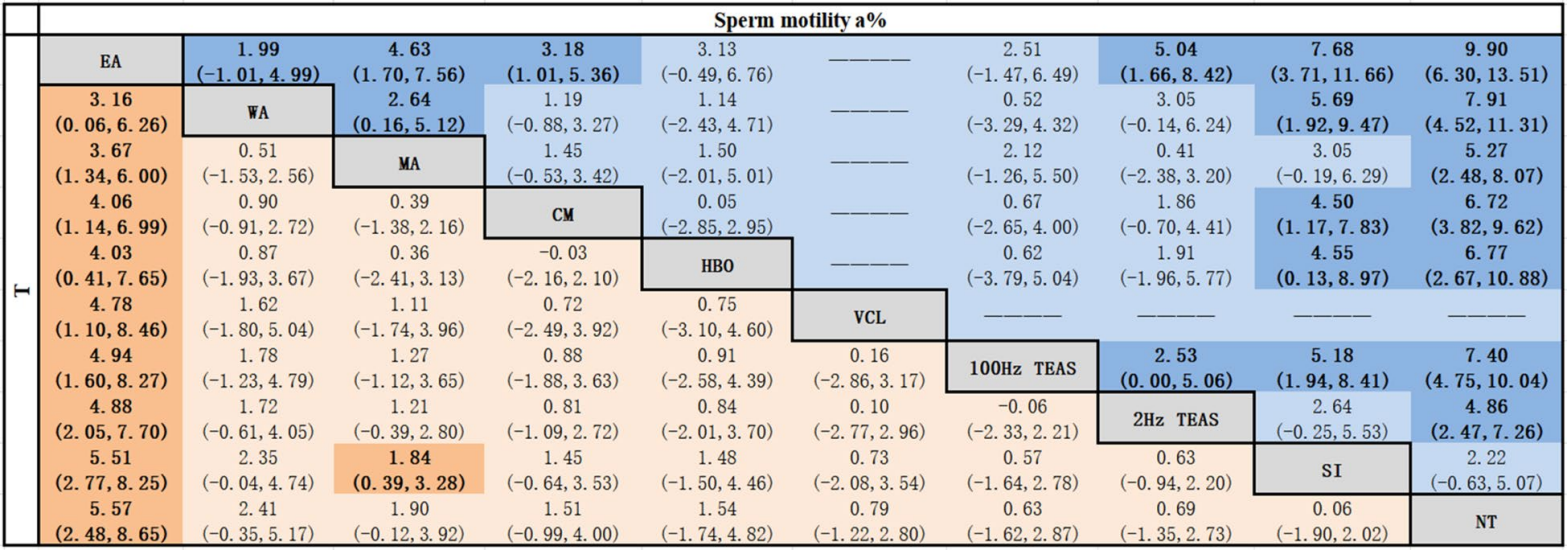
Network meta-analysis of sperm motility a% and T [MD (95% CI)]

Bold values are statistically significant; Tables 2, 3, 4, 5 are the same.

##### FSH

In terms of FSH reduction, 100 Hz TEAS was better than SI [MD = − 0.81, 95% CI (− 0.37, − 0.25)] and NT [MD = − 0.99, 95% CI (− 1.61, − 0.37)]. HBO, EA, WA, CM, 2 Hz TEAS, MA were better than SI, VCL, and NT. Compared with 100 Hz TEAS, HBO, EA, WA, CM, 2 Hz TEAS and MA were more effective at reducing FSH. All of the abovementioned differences were statistically significant (*P* < 0.05), as shown in Table 5.

**Table 5:**
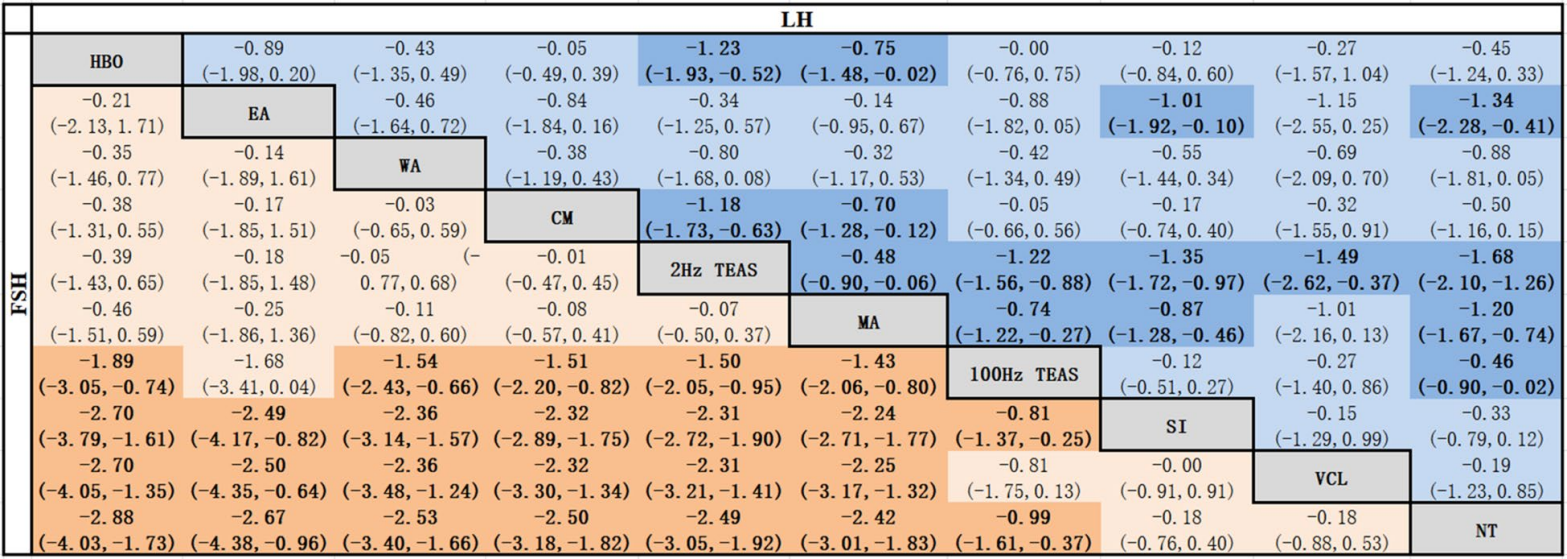
Network meta-analysis of FSH and LH [MD (95% CI)]

Bold values are statistically significant; Tables 2, 3, 4, 5 are the same.

**Table 6:** Ranking of SUCRA probabilities for each outcome indicator

Intervention	Total effective rate		Sperm concentration		Sperm motility a + b%		Sperm motility a%	
	SUCRA	RANK	SUCRA	RANK	SUCRA	RANK	SUCRA	RANK
WA	80.1	1	93.4	1	65.8	5	78.1	2
EA	75.2	2	63.7	5	82.0	1	96.6	1
2 Hz TEAS	67.7	4	56.3	7	74.1	3	33.2	7
100 Hz TEAS	62.9	6	30.2	8	75.0	2	70.5	3
MB	75.0	3	83.7	2	21.5	9	–	–
MA	28.8	9	63.8	4	71.4	4	37.0	6
VCL	44.0	8	16.7	9	30.4	8	–	–
HBO	64.6	5	65.9	3	64.5	6	60.6	4
CM	44.2	7	62.4	6	47.6	7	60.2	5
SI	10.3	10	13.5	10	12.9	10	12.9	8
NT	0.2	11	0.4	11	4.8	11	0.8	9
Intervention	Adverse reaction		FSH		LH		T	
	SUCRA	RANK	SUCRA	RANK	SUCRA	RANK	SUCRA	RANK
WA	68.8	2	71.4	3	62.8	4	75.8	2
EA	60.8	4	74.5	2	82.7	2	99.4	1
2 Hz TEAS	38.1	8	68.9	5	96.8	1	35.2	8
100 Hz TEAS	38.8	7	33.1	7	39.3	6	35.3	7
MB	20.4	11	–	–	–	–	–	–
MA	27.7	10	63.5	6	78.6	3	68.0	3
VCL	58.2	5	13.6	9	24.9	9	40.3	6
HBO	55.2	6	85.4	1	37.0	7	54.2	5
CM	31.0	9	69.5	4	41.6	5	55.6	4
SI	62.9	3	13.7	8	28.8	8	18.3	9
NT	88.0	1	6.6	10	7.6	10	17.8	10

##### LH

Compared to NT, 2 Hz TEAS [MD = − 0.81, 95% CI (− 0.37, − 0.25)], EA [MD = − 0.81, 95% CI (− 0.37, − 0.25)], MA [MD = − 0.81, 95% CI (− 0.37, − 0.25)], and 100 Hz TEAS [MD = − 0.81, 95% CI (− 0.37, − 0.25)] were better at reducing LH. 2 Hz TEAS was better than MA, CM, 100 Hz TEAS, HBO, SI, VCL, and NT. MA was better than CM, 100 Hz TEAS, HBO, SI, and NT. EA was a better intervention than SI and NT. All of the abovementioned differences were statistically significant (*P* < 0.05), as shown in Table 5.

#### SUCRA probability ranking

##### Total effective rate

WA was probably the most effective intervention for improving the total effective rate (80.1%), followed by EA (75.2%), MB (75.0%), 2 Hz TEAS (64.7%), HBO (64.6%), 100 Hz TEAS (62.9%), CM (44.2%), VCL (44.0%) MA (28.8%), SI (10.3%), and NT (0.2%).

##### Sperm concentration

WA was perhaps the most effective intervention for increasing the sperm concentration (93.4%), followed by MB (83.7%), HBO (65.9%), MA (63.8%), EA (63.7%), CM (62.4%), 2 Hz TEAS (56.3%) > 100 Hz TEAS (30.2%), VCL (16.7%), SI (13.5%), and NT (0.4%).

##### Sperm viability a%

EA was probably the most effective intervention for increasing the sperm viability a% (96.6%), followed by WA (78.1%), 100 Hz TEAS (70.5%), HBO (60.6%), CM (60.2%), MA (37.0%), 2 Hz TEAS (33.2%), SI (12.9%), and NT (0.8%).

##### Sperm viability a + b%

EA was probably the most effective intervention for improving the sperm viability a + b% (82.0%), followed by 100 Hz TEAS (75.0%), 2 Hz TEAS (74.1%), MA (71.4%), WA (65.8%), HBO (64.5%), CM (47.6%), VCL (30.4%), MB (21.5%), SI (12.9%) and NT (4.8%).

##### Adverse reaction

NT was perhaps the safest intervention (88.0%), followed by WA (68.8%), SI (62.9%), EA (60.8%), VCL (58.2%), HBO (55.2%), 100 Hz TEAS (38.8%), 2 Hz TEAS (38.1%), CM (31.0%), MA (27.7%), and MB (20.4%).

##### FSH

HBO was perhaps the most effective intervention for reducing FSH (85.4%) > EA (74.5%) > WA (71.4%) > CM (69.5%) > 2 Hz TEAS (68.9%) > MA (63.5%) > 100 Hz TEAS (33.1%) > SI (13.7%) > VCL (13.6%) > NT (6.6%).

##### LH

2 Hz TEAS was probably the most effective intervention for reducing LH (96.8%), followed by EA (82.7%), MA (78.6%), WA (62.8%), CM (41.6%), 100 Hz TEAS (39.3%), HBO (37.0%), SI (28.8%), VCL (24.9%), and NT (7.6%).

##### T

EA was probably the most effective intervention for increasing T (99.4%), followed by WA (75.8%), MA (68.0%), CM (55.6%), HBO (54.2%), VCL (40.3%), 100 Hz TEAS (35.3%), 2 Hz TEAS (35.2%), SI (18.3%), and NT (17.8%).

The SUCRA values and ranking results for each outcome indicator are shown in Table 6, with higher SUCRA values suggesting more effective or safer interventions.

### Publication bias

**Fig. 9:**
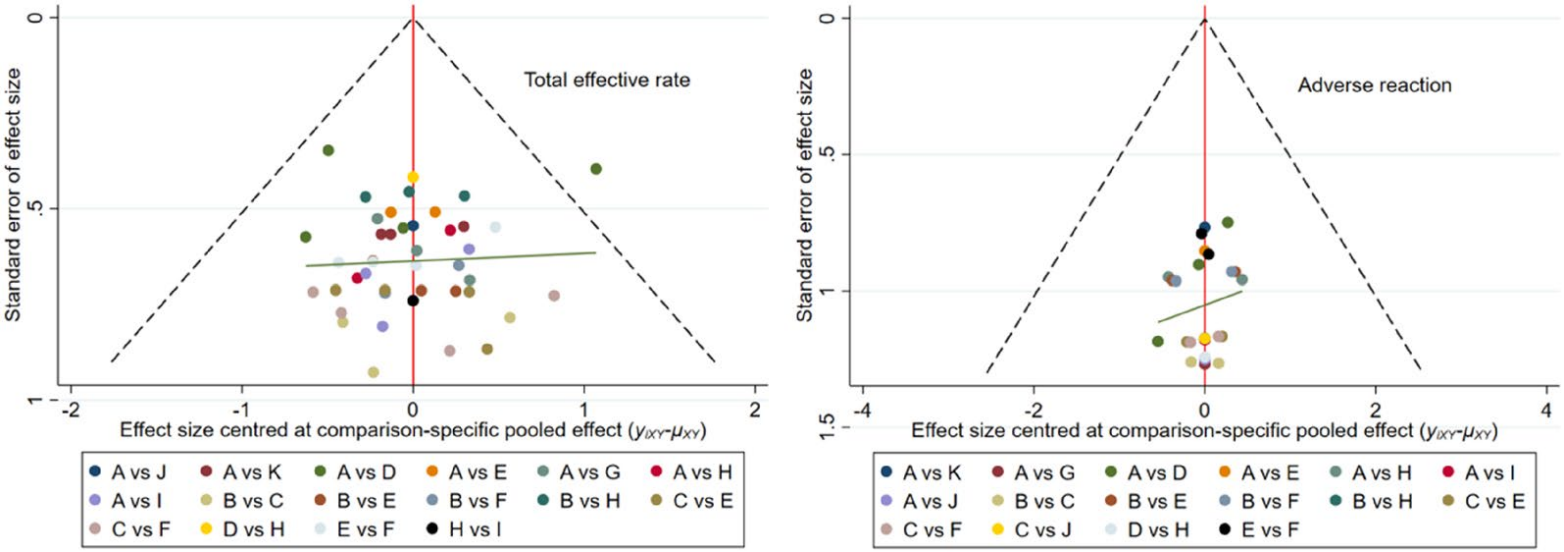
Comparison-corrected funnel plot of the total effective rate and adverse reactions A: CM, B: SI, C: NT, D: EA, E: 2 Hz TEAS, F: 100 Hz TEAS, G: MB, H: MA, I: WA, J: VCL, K: HBO

Stata 16.0 was used to test for small-sample effects for each outcome indicator, including total effective rate, sperm concentration, sperm motility (a/a + b levels), incidence of adverse events, T, FSH and LH. Stata was also used to produce “comparison-corrected” funnel plots. The results show that the funnel plots for the total effective rate and the incidence of adverse events have good symmetry, suggesting that the quality of the included studies is high and the possibility of publication bias is low (see Fig. 9). The funnel plots for the remaining outcome indicators have poor symmetry, suggesting that there may be some publication bias (see Additional file 1: Figures S2, S3, S4).

### Sensitivity analysis

To test the reliability and stability of this network meta-analysis, 4 sensitivity analyses were performed using Stata 16.0. First, 4 papers reported cases of oligoasthenospermia complicated with varicocele. To reduce the effect of complications on this outcome, these 4 [55–58] papers were excluded, and then sensitivity analyses were performed on the pre- and postexclusion papers. Second, the three papers [27, 44, 60] that were evaluated as high risk in terms of literature quality were excluded, and then sensitivity analyses were performed before and after exclusion. Third, considering that the RCTS with a small sample size may affect the accuracy of the results, 10 literatures [34, 42, 46, 49–51, 55, 57, 61, 62] with a sample size less than 60 were excluded for sensitivity analysis. Finally, the treatment duration of 5 articles [35, 36, 49, 59, 60] is less than or equal to 1 month, which may cause potential bias. Therefore, these 5 articles were deleted for sensitivity analysis. This time, sensitivity analyses were performed only on the effectiveness indicators (total effective rate, sperm concentration, sperm viability a + b%) and safety indicators (incidence of adverse reactions). The results show that there is little difference between the results before and after the exclusion of four sensitivity analyses, indicating that the quality of the literature is good and that this network meta-analysis is reliable and stable. The results of the sensitivity analysis are shown in Additional file Pages 11–18.

## Discussion

Up to now, neither meta-analysis nor systematic review on the effect and safety of nonpharmacological strategies in the treatment of OAT have been reported. In this meta-analysis, 38 RCTs with a total of 3080 patients were included to assess the effect and safety of nonpharmacological strategies in patients with OAT.In the current study, we used the total effective rate, sperm concentration and sperm viability a/a + b% as indicators of efficacy and the incidence of adverse events as an indicator of safety to investigate the advantages of each intervention on each outcome indicator. The top three interventions in terms of overall effectiveness were MA, EA, MB. WA, MB, HBO were the top three interventions in terms of increasing sperm concentration. EA, WA, 100 Hz were the top three interventions in terms of increasing sperm motility a%. EA, 100 Hz TEAS, 2 Hz TEAS were the top three interventions in terms of increasing sperm motility a + b%. NT, WA, SI, EA, and VCL were the top five interventions in terms of reducing the incidence of adverse events. After analysing the various outcome indicators in this study, it was found that there was a wide variation in the optimal ranking of the treatments in the different outcome indicators, making it difficult to choose the optimal option; for example, MB ranked highly in improving overall efficiency and sperm concentration but had the worst safety profile.

In-depth analysis of the indicators revealed that the nonpharmacological treatments included in this study were superior to both SI and NT in terms of improving effectiveness, with warm acupuncture and electroacupuncture ranking highly and consistently in terms of efficacy, as well as being safer. Warm acupuncture was observed to be good at increasing sperm concentration, and electroacupuncture was found to be the most likely effective intervention for improving sperm viability. Warm acupuncture is a complementary alternative therapy that combines acupuncture and moxibustion, fully integrating the “opening” of acupuncture and the “warming” of moxibustion to benefit the essence by warming the meridians and running the qi and blood [63]. Modern studies have shown that warm needling improves the internal environment of the testis, intervenes in oxidative stress damage, and protects sperm membrane structure and function, thereby improving spermatogenic function [64]. Experiments have also confirmed that warm needling elevates sperm concentration and improves seminal plasma neutral alpha-glucosidase and seminal plasma zinc levels [65].

Electroacupuncture combines Chinese acupuncture with modern medicine bioelectricity, which, by enhancing nerve conduction, can propel sperm movement and keep its movement pathways unobstructed. Studies have shown that electroacupuncture promotes the release of β-endorphins and increases the acrosome response of sperm, which in turn enhances sperm motility [66]. In addition, electroacupuncture can improve sperm motility by increasing the level of SOD activity and scavenging excess oxygen free radicals in the body [67].

Modern medicine suggests that spermatogenesis, maturation and motility are regulated by the reproductive hormones FSH, LH and T [68]. We analysed these three hormones as outcome indicators. The results found that warm and electroacupuncture, which ranked high in the effectiveness index, also improved FSH, LH and T more effectively, with some positive correlation, which may be the underlying mechanism of action. However, there is also a certain negative correlation, e.g., 2 Hz TEAS improves LH the best, but all of them are poorly ranked in terms of effectiveness and, therefore, still need to be studied in a large number of experiments. Studies have shown that 15–20% of patients with OAT have varying degrees of varicocele, which has been shown to be an important influencing factor on semen quality in patients with oligoasthenospermia [69, 70]. Four included trials examine patients with varicocele to investigate changes in semen quality after varicocele removal. The results found that all indicators improved in patients after surgery but were lower in the ranking, suggesting that it is difficult to obtain significant clinical outcomes with surgery alone and that other interventions could be applied postoperatively if necessary. Therefore, the application of the above interventions should be tailored to the characteristics and condition of the patient, and the probability ranking results are for clinicians’ reference only.

There are also some limitations to this study. (i) Due to the relatively strict inclusion and exclusion criteria, RCTs with shock wave, five-animal exercise and laser methods were not included, and therefore, no statistical analysis of these therapies was conducted. (ii) The sample size of the included studies was mixed, and only a few papers mentioned the follow-up process. (iii) None of the papers included in this study had published pretrial protocols, which may have led to selective reporting bias. (iv) Adverse reactions cannot be broken down and are very widespread, which may result in potential bias.

In summary, nonpharmacological treatments for oligoasthenospermia have good clinical efficacy. Warm acupuncture is good at boosting sperm concentration, and electroacupuncture can be given priority for treatment when patients have low sperm motility as the main symptom. When varicocele is present, it should be removed surgically and then treated with other interventions as appropriate. Due to the limitations of study quality and sample size, additional large-sample, multicentre and high-quality clinical trials are needed to supplement the validation note with a view to providing stronger evidence to support nonpharmacological therapies for the treatment of oligoasthenospermia.

## Supplementary Information

**Additional file 1: Table S1** Introduction to different Interventions. **Table S2** Search Strategies of Pubmed. **Table S3** Node-splitting test of total effective rate. **Table S4** Node-splitting test of sperm concentration. **Table S5** Node-splitting test of sperm motility a%. **Table S6** Node-splitting test of sperm motility a+b%. **Table S7** Node-splitting test of adverse reaction. **Table S8** Node-splitting test of FSH. **Table S9** Node-splitting test of LH. **Table S10** Node-splitting test of T. **Table S11** Ranking of SUCRA probabilities for each outcome indicator. **Table S12** Network meta-analysis of total effective rate and adverse reaction [RR (95%CI)]. **Table S13** Network meta-analysis of sperm concentration and sperm motility a+b% [MD (95%CI)]. **Table S14** Ranking of SUCRA probabilities for each outcome indicator. **Table S15** Network meta-analysis of total effective rate and adverse reaction［RR (95%CI)]. **Table S16** Network meta-analysis of sperm concentration and sperm motility a+b% [OR (95%CI)]. **Table S17** Ranking of SUCRA probabilities for each outcome indicator. **Table S18** Network meta-analysis of total effective rate and adverse reaction [RR (95%CI)]. **Table S19** Network meta-analysis of sperm concentration and sperm motility a+b% [OR (95%CI)]. **Table S20** Ranking of SUCRA probabilities for each outcome indicator. **Table S21** Network meta-analysis of total effective rate and adverse reaction [RR (95%CI)]. **Table S22** Network meta-analysis of sperm concentration and sperm motility a+b% [OR (95%CI)]. **Table S23** Specific circumstances of the occurrence of adverse reactions. Table S24 Protocol amendment. **Fig. S1** Risk of bias summary. **Fig. S2** “Compare-corrected” funnel plot of sperm concentration and sperm motility a+b%. **Fig. S3** “Compare-corrected” funnel plot of FSH、LH and T. **Fig. S4** Compare-corrected” funnel plot of sperm motility a%. **Fig. S5** Evidence network diagram for each outcome indicator. **Fig. S6** Evidence network diagram for each outcome indicator. **Fig. S7** Evidence network diagram for each outcome indicator. **Fig. S8** Evidence network diagram for each outcome indicator. **Fig. S9** GRADE Assessment of Quality of Evidence.

## Data Availability

The original contributions presented in the study are included in the article/Additional file 1, and further inquiries can be directed to the corresponding author.

## Abbreviations

OAT: Oligoasthenospermia
RCTs: Randomized controlled trials
WHO: World Health Organization
FSH: Follicle-stimulating hormone
LH: Luteinizing hormone
T: Testosterone
EA: Electroacupuncture
TEAS: Transcutaneous electrical acupoint stimulation
MB: Moxibustion
MA: Manual acupuncture
WA: Warming acupuncture
VCL: Varicocelectomy
HBO: Hyperbaric oxygen
CM: Conventional medicine
SI: Sham intervention
NT: No treatment
