# Supplementary material for: Efficacy and safety of nonpharmacological strategies for the treatment of oligoasthenospermia: a systematic review and Bayesian network meta-analysis
Source: Eur J Med Res. 2023 Jan 4;28:6. doi: 10.1186/s40001-022-00968-6 (PMC9811722; doi:10.1186/s40001-022-00968-6)
Supplement: Supplementary file 1 — Additional file 1: Table S1 Introduction to different Interventions. Table S2 Search Strategies of Pubmed. Table S3 Node-splitting test of total effective rate. Table S4 Node-splitting test of sperm concentration. Table S5 Node-splitting test of sperm motility a%. Table S6 Node-splitting test of sperm motility a+b%. Table S7 Node-splitting test of adverse reaction. Table S8 Node-splitting test of FSH. Table S9 Node-splitting test of LH. Table S10 Node-splitting test of T. Table S11 Ranking of SUCRA probabilities for each outcome indicator. Table S12 Network meta-analysis of total effective rate and adverse reaction [RR (95%CI)]. Table S13 Network meta-analysis of sperm concentration and sperm motility a+b% [MD (95%CI)]. Table S14 Ranking of SUCRA probabilities for each outcome indicator. Table S15 Network meta-analysis of total effective rate and adverse reaction［RR (95%CI)]. Table S16 Network meta-analysis of sperm concentration and sperm motility a+b% [OR (95%CI)]. Table S17 Ranking of SUCRA probabilities for each outcome indicator. Table S18 Network meta-analysis of total effective rate and adverse reaction [RR (95%CI)]. Table S19 Network meta-analysis of sperm concentration and sperm motility a+b% [OR (95%CI)]. Table S20 Ranking of SUCRA probabilities for each outcome indicator. Table S21 Network meta-analysis of total effective rate and adverse reaction [RR (95%CI)]. Table S22 Network meta-analysis of sperm concentration and sperm motility a+b% [OR (95%CI)]. Table S23 Specific circumstances of the occurrence of adverse reactions. Table S24 Protocol amendment. Fig. S1 Risk of bias summary. Fig. S2 “Compare-corrected” funnel plot of sperm concentration and sperm motility a+b%. Fig. S3 “Compare-corrected” funnel plot of FSH、LH and T. Fig. S4 Compare-corrected” funnel plot of sperm motility a%. Fig. S5 Evidence network diagram for each outcome indicator. Fig. S6 Evidence network diagram for each outcome indicator. Fig. S7 Evidence network diagram for each outcome i [file 40001_2022_968_MOESM1_ESM.docx]

**Efficacy and safety of nonpharmacological strategies for the treatment of oligoasthenospermia: A systematic review and Bayesian network meta-analysis**

Zhen Wang, Ziyang Zhou, Lijuan Zhang, Xujie Li, Miaoxiu Li, Yankun Pan, Tiyong Jiao , Xiaoyun Shi,Qing Liu,Congan Wang, Yongquan Wang

**Additional file**

|  | |  |
| --- | --- | --- |
| **Introduction to different Interventions** | **Table 1** | **Page 2** |
| **Search Strategies of Pubmed** | **Table 2** | **Page 3** |
| **Risk of bias summary** | **Fig.1** | **Page 4** |
| **“Compare-corrected” funnel plot** | **Fig.2-4** | **Page 5-6** |
| **Node-splitting test** | **Table 3-10** | **Page7-10** |
| **Results of Sensitivity analyses** | **Table 11-22** | **Page11-18** |
|  | **Fig.5-8** |  |
| **GRADE Assessment of Quality of Evidence** | **Fig.9** | **Page 19** |
| **The situation of the adverse reactions** | **Table 23** | **Page 20** |
| **Protocol amendment** | **Table 24** | **Page 21** |

**Table S1 Introduction to different Interventions**

| **Interventions** | **Abbreviation** | **Description** |
| --- | --- | --- |
| **Warming acupuncture** | **WA** | **WA is a method of treating disease by combining acupuncture with moxibustion, using moxa and other methods to heat the shank of the needles pierced into the body. Operation: After the needles have been inserted into the acupuncture point and the appropriate complementary and diaphoretic techniques have been given, moxa is wrapped around the end of the needles or a section of moxa stick about 1 to 2 cm long is inserted into the shank of the needles and ignited for moxibustion during the moxibustion process. After burning out, remove the ashes of the moxa and reload for further moxibustion. After the prescribed number of strokes is completed, the needles can be removed.** |
| **Manual acupuncture** | **MA** | **A needle is inserted into a specific acupoint at a certain angle according to the theory of traditional Chinese medicine. Acupuncture techniques such as twisting and lifting are used to stimulate specific parts of the body to treat diseases.** |
| **Transcutaneous electrical acupoint stimulation** | **TEAS** | **Electrode pads are applied to specific acupuncture points and energised with a trace current close to the body's bioelectricity to prevent and treat disease, this study is divided into 2Hz and 100Hz groups depending on the frequency.** |
| **Electroacupuncture** | **EA** | **EA is a method of preventing and treating disease by combining needle and electrical stimulation by passing a trace current close to the body's bioelectricity through the needle tool after the needle has been inserted into the acupoint to obtain Qi.** |
| **Moxibustion** | **MB** | **MB is a treatment method that uses moxa leaves to make moxa sticks, moxa pillars, which produce moxa heat to stimulate acupuncture points or specific areas of the body for the purpose of preventing and treating disease.** |
| **Varicocelectomy** | **VCL** | **A treatment method that uses a surgical procedure to minimally invasively ligate varicose veins for the purpose of preventing and treating disease.** |
| **Hyperbaric oxygen** | **HBO** | **A method of treating disease by using a hyperbaric chamber where the patient is placed under high pressure (above normal pressure) and breathing pure or highly concentrated oxygen.** |
| **Conventional medicine** | **CM** | **All drugs must be approved by the US Food and Drug Administration or the State Drug Administration of China and conform to the guidelines or expert consensus recommendations for the treatment of oligozoospermia,including western medicine and Chinese patent medicine.** |
| **Sham intervention** | **SI** | **It is a sham intervention that has no therapeutic effect and has a substitution and comforting effect. This study is sham acupuncture and mock TEAS.** |
| **No treatment** | **NT** | **No treatment for patients with oligoasthenospermia.** |

**Table S2 Search Strategies of Pubmed**

**
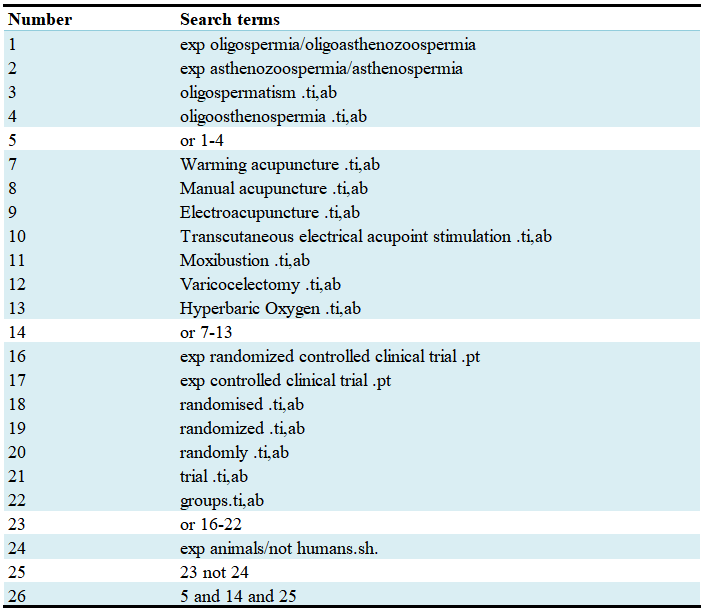
**

**Fig.S1 Risk of bias summary**


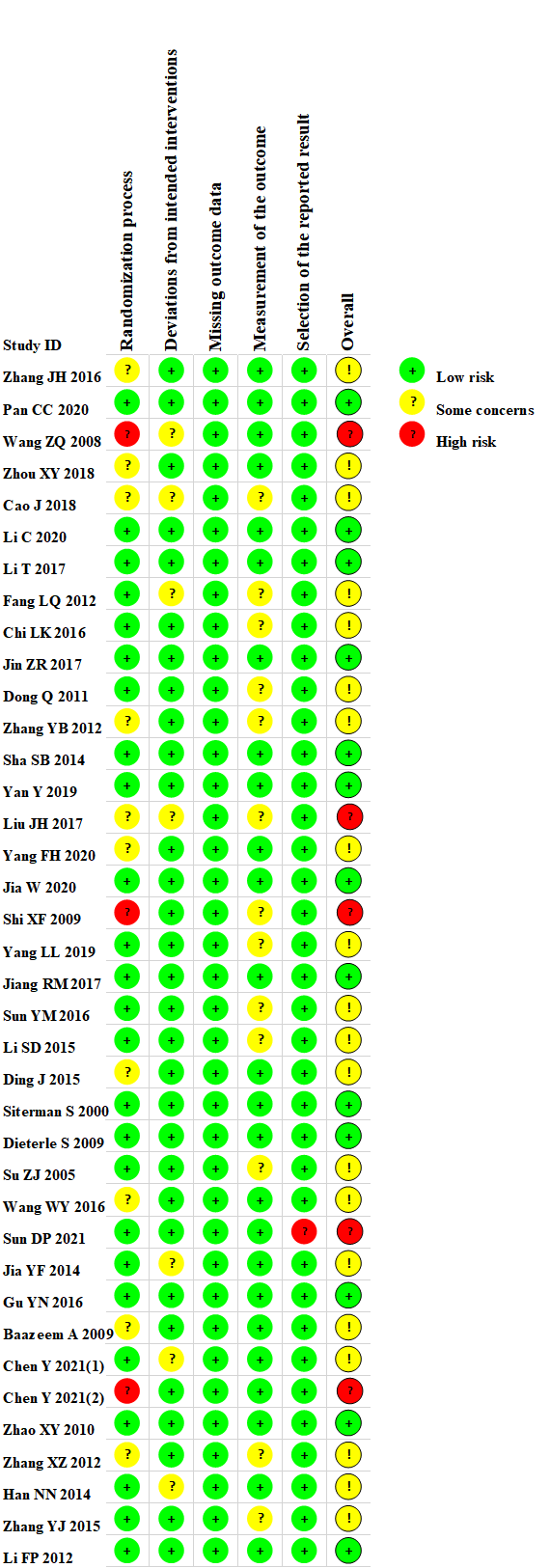

注：A:CM B:SI C:NT D:EA E:2HzTEAS F:100HzTEAS G:MB H:MA I:WA J:VCL K:HBO

**Fig. S2 “Compare-corrected” funnel plot of sperm concentration and sperm motility a+b%**

注：A:CM B:SI C:NT D:EA E:2HzTEAS F:100HzTEAS G:MA H:WA I:VCL J:HBO

**Fig. S3 “Compare-corrected” funnel plot of FSH、LH and T**

注：A:CM B:SI C:NT D:EA E:2HzTEAS F:100HzTEAS G:MA H:WA I:HBO

**Fig. S4 Compare-corrected” funnel plot of sperm motility a%**

**Table S3 Node-splitting test of total effective rate**

| Side | Direct | | Indirect | | Difference | | P |
| --- | --- | --- | --- | --- | --- | --- | --- |
|  | Coef. | Std. Err. | Coef. | Std. Err. | Coef. | Std. Err. |  |
| CM vs EA | 0.4932 | 0.2371 | 0.0886 | 0.5724 | 0.4046 | 0.6196 | 0.514 |
| CM vs 2Hz TEAS | -0.0000 | 0.3792 | 0.3737 | 0.5872 | -0.3738 | 0.6990 | 0.593 |
| CM vs MA | -0.3090 | 0.4488 | -0.2058 | 0.3851 | -0.1031 | 0.5948 | 0.862 |
| CM vs WA | -0.3090 | 0.4488 | -0.2058 | 0.3851 | -0.1031 | 0.5948 | 0.862 |
| SI vs NT | -0.5787 | 0.4861 | -0.5281 | 0.8994 | -0.0506 | 1.0200 | 0.960 |
| SI vs 2Hz TEAS | 1.3962 | 0.4063 | 0.9684 | 0.5259 | 0.4278 | 0.6550 | 0.514 |
| SI vs 100Hz TEAS | 1.2072 | 0.4101 | 0.6791 | 0.8562 | 0.5280 | 0.9509 | 0.579 |
| SI vs MA | 0.8170 | 0.2849 | 1.1905 | 0.6384 | -0.3735 | 0.6991 | 0.593 |
| NT vs 2Hz TEAS | 1.8415 | 0.3808 | 1.0939 | 1.3829 | 0.7476 | 1.3981 | 0.593 |
| EA vs MA | -0.4682 | 0.4517 | -0.8728 | 0.4240 | 0.4046 | 0.6196 | 0.514 |
| 2Hz TEAS vs 100Hz | -0.1673 | 0.3188 | 0.5803 | 1.3642 | -0.7476 | 1.3981 | 0.593 |
| MA vs WA | 1.0147 | 0.7472 | 0.6832 | 0.5622 | 0.3315 | 0.9246 | 0.720 |

**Table S4 Node-splitting test of sperm concentration**

| Side | Direct | | Indirect | | Difference | | P |
| --- | --- | --- | --- | --- | --- | --- | --- |
|  | Coef. | Std. Err. | Coef. | Std. Err. | Coef. | Std. Err. |  |
| CM vs EA | -0.4112 | 1.0783 | 4.9876 | 3.5613 | -5.3988 | 3.7211 | 0.147 |
| CM vs 2Hz TEAS | -0.8536 | 1.6008 | 0.1766 | 2.1737 | -1.0302 | 2.6994 | 0.703 |
| CM vs MA | 0.3785 | 1.3633 | -0.5943 | 1.9606 | 0.9729 | 2.3935 | 0.684 |
| CM vs WA | 2.6474 | 1.5090 | 2.1813 | 4.8714 | 0.4661 | 5.0896 | 0.927 |
| CM vs VCL | -6.0600 | 2.1983 | -8.1383 | 2.3697 | 2.0783 | 3.2324 | 0.520 |
| SI vs NT | -3.7029 | 1.6890 | -0.0122 | 2.6906 | -3.6906 | 3.1841 | 0.246 |
| SI vs 2Hz TEAS | 7.0959 | 1.7580 | 7.3940 | 2.6362 | -0.2980 | 3.1543 | 0.925 |
| SI vs MA | 9.8391 | 2.1359 | 5.6733 | 2.0971 | 4.1658 | 2.9933 | 0.164 |
| NT vs 2Hz TEAS | 10.6739 | 1.5335 | 7.2939 | 2.5851 | 3.3799 | 3.0065 | 0.261 |
| NT vs 100Hz TEAS | 6.9731 | 1.5552 | -1.9255 | 5.0781 | 8.8987 | 5.3274 | 0.095 |
| NT vs MA | 7.0878 | 2.9034 | 11.5524 | 1.7623 | -4.4645 | 3.3907 | 0.188 |
| NT vs VCL | 2.71012 | 1.6781 | 4.8634 | 2.7723 | -2.1532 | 3.2338 | 0.506 |
| EA vs MA | -4.4200 | 3.3860 | 0.9789 | 1.5433 | -5.3989 | 3.7212 | 0.147 |
| 2Hz TEAS vs 100Hz | -3.7104 | 1.5350 | -1.6499 | 5.1996 | -2.0605 | 5.3988 | 0.703 |
| MA vs WA | 1.7251 | 2.3889 | 3.2885 | 2.2602 | -1.5634 | 3.2798 | 0.634 |

**Table S5 Node-splitting test of sperm motility a%**

| Side | Direct | | Indirect | | Difference | | P |
| --- | --- | --- | --- | --- | --- | --- | --- |
|  | Coef. | Std. Err. | Coef. | Std. Err. | Coef. | Std. Err. |  |
| CM vs EA | -1.2300 | 1.6699 | -3.3134 | 2.4288 | 2.0834 | 2.9475 | 0.480 |
| CM vs 2Hz TEAS | -1.6777 | 1.2335 | -0.4671 | 2.3809 | -1.2106 | 2.6836 | 0.652 |
| CM vs MA | 0.6817 | 0.9781 | 7.0766 | 3.5908 | -6.3948 | 3.7580 | 0.089 |
| SI vs 2Hz TEAS | 1.4511 | 1.8040 | 4.9647 | 2.5367 | -3.5135 | 3.2108 | 0.274 |
| SI vs 100Hz TEAS | 4.8834 | 2.2803 | 5.5802 | 3.0527 | -0.6968 | 4.0793 | 0.864 |
| SI vs MA | 2.0000 | 2.8879 | 3.6134 | 2.0961 | -1.6134 | 3.5685 | 0.651 |
| NT vs 2Hz TEAS | 4.2621 | 1.4106 | 7.4757 | 2.9849 | -3.2135 | 3.3053 | 0.331 |
| NT vs 100Hz TEAS | 7.0594 | 1.4399 | 13.5652 | 5.8218 | -6.5058 | 5.9754 | 0.276 |
| NT vs MA | 7.0000 | 2.1614 | 3.7465 | 2.0628 | 3.2534 | 2.9878 | 0.276 |
| 2Hz TEAS vs 100Hz | 2.7439 | 1.3976 | -1.4229 | 5.7774 | 4.1669 | 5.8951 | 0.480 |
| MA vs WA | 3.9675 | 1.7001 | 0.9119 | 1.9691 | 3.0555 | 2.6054 | 0.241 |

**Table S6 Node-splitting test of sperm motility a+b%**

| Side | Direct | | Indirect | | Difference | | P |
| --- | --- | --- | --- | --- | --- | --- | --- |
|  | Coef. | Std. Err. | Coef. | Std. Err. | Coef. | Std. Err. |  |
| CM vs EA | 4.0858 | 3.4279 | 3.1414 | 5.5049 | 0.9444 | 6.4851 | 0.884 |
| CM vs 2Hz TEAS | 6.5069 | 2.8770 | -1.6529 | 3.0528 | 8.1599 | 4.1945 | 0.052 |
| CM vs MA | -0.3052 | 2.7002 | 5.5167 | 2.9403 | -5.8219 | 3.9959 | 0.145 |
| CM vs WA | 1.3433 | 2.5195 | 7.2732 | 9.2403 | -5.9299 | 9.5919 | 0.536 |
| CM vs VCL | -4.8100 | 4.2869 | -1.5556 | 4.0098 | -3.2543 | 5.8700 | 0.579 |
| SI vs NT | -2.6643 | 2.5890 | 0.2700 | 3.7818 | -2.9343 | 4.5828 | 0.522 |
| SI vs 2Hz TEAS | 8.8798 | 2.2692 | 11.5011 | 4.4952 | -2.6213 | 5.0373 | 0.603 |
| SI vs 100Hz TEAS | 7.4032 | 2.4613 | 17.2919 | 4.6072 | -9.8887 | 5.2237 | 0.058 |
| SI vs MA | 11.8704 | 2.8382 | 5.1052 | 3.4532 | 6.7652 | 4.4682 | 0.130 |
| NT vs 100Hz TEAS | 10.7380 | 2.1742 | 20.2808 | 8.5085 | -9.5427 | 8.7785 | 0.277 |
| NT vs MA | 13.9999 | 4.6076 | 9.7613 | 2.7546 | 4.2386 | 5.3682 | 0.430 |
| NT vs VCL | 6.2249 | 2.8618 | 2.9826 | 5.1289 | 3.2423 | 5.8715 | 0.581 |
| EA vs MA | -0.9000 | 5.0530 | -1.8441 | 4.0650 | 0.9440 | 6.4852 | 0.884 |
| 2Hz TEAS vs MA | -1.8933 | 4.6701 | 0.2469 | 2.6083 | -2.1403 | 5.3438 | 0.689 |
| MA vs WA | 0.7021 | 4.6087 | -1.4696 | 3.8127 | 2.1718 | 5.9811 | 0.717 |

**Table S7 Node-splitting test of adverse reaction**

| Side | Direct | | Indirect | | Difference | | P |
| --- | --- | --- | --- | --- | --- | --- | --- |
|  | Coef. | Std. Err. | Coef. | Std. Err. | Coef. | Std. Err. |  |
| CM vs EA | -0.6551 | 0.5186 | -0.5997 | 1.3875 | -0.0554 | 1.4813 | 0.970 |
| CM vs 2Hz TEAS | -6.0511 | 0.8539 | -0.4935 | 1.2016 | 0.4935 | 1.4741 | 0.738 |
| CM vs MA | 0.0558 | 0.6747 | 0.2880 | 1.0129 | -0.2321 | 1.2171 | 0.849 |
| CM vs VCL | -0.7248 | 1.2504 | -0.5395 | 1.5872 | -0.1853 | 2.0206 | 0.927 |
| SI vs NT | -0.9791 | 0.8889 | -0.6204 | 2.1261 | -0.3586 | 2.2904 | 0.876 |
| SI vs 2Hz TEAS | 0.5072 | 0.6679 | 1.0882 | 1.5515 | -0.5810 | 1.6854 | 0.730 |
| SI vs 100Hz TEAS | 0.5465 | 0.6690 | 1.5398 | 3.2027 | -0.9933 | 3.3040 | 0.764 |
| SI vs MA | 1.1402 | 1.1726 | 0.6435 | 1.1637 | 0.4966 | 1.6520 | 0.764 |
| NT vs 2Hz TEAS | 1.4402 | 0.8253 | 1.9621 | 1.8099 | -0.5219 | 1.9577 | 0.790 |
| NT vs 100Hz TEAS | 1.5006 | 0.8318 | 1.8702 | 3.8654 | -0.3695 | 4.0414 | 0.927 |
| NT vs VCL | 1.0986 | 1.1737 | 0.9135 | 1.6449 | 0.1851 | 2.0208 | 0.927 |
| EA vs MA | 0.7366 | 1.2423 | 0.7920 | 0.8067 | -0.0554 | 1.4813 | 0.970 |
| 2Hz TEAS vs 100Hz | 0.0383 | 0.5838 | -0.9488 | 2.8901 | 0.9871 | 2.9482 | 0.738 |

**Table S8 Node-splitting test of FSH**

| Side | Direct | | Indirect | | Difference | | P |
| --- | --- | --- | --- | --- | --- | --- | --- |
|  | Coef. | Std. Err. | Coef. | Std. Err. | Coef. | Std. Err. |  |
| CM vs 2Hz TEAS | 0.1199 | 0.2857 | -0.2179 | 0.4175 | 0.3379 | 0.5059 | 0.504 |
| CM vs MA | -0.0670 | 0.3319 | 0.2603 | 0.3716 | -0.3274 | 0.4934 | 0.507 |
| CM vs WA | -0.0692 | 0.3205 | 0.6043 | 1.0080 | -0.6735 | 1.0118 | 0.506 |
| SI vs NT | 0.3568 | 0.3659 | -0.1586 | 0.4974 | 0.5155 | 0.6137 | 0.401 |
| SI vs 2Hz TEAS | -2.3538 | 0.2181 | -1.7430 | 0.7769 | -0.6108 | 0.8055 | 0.448 |
| SI vs 100Hz TEAS | -0.8000 | 0.3233 | -0.8565 | 0.7360 | 0.0565 | 0.8300 | 0.946 |
| SI vs MA | -2.3013 | 0.2836 | -2.1029 | 0.4467 | -0.1984 | 0.5288 | 0.708 |
| NT vs 2Hz TEAS | -2.7855 | 0.3531 | -1.9248 | 0.4800 | -0.8607 | 0.5877 | 0.143 |
| NT vs 100Hz TEAS | -1.1700 | 0.3356 | 0.8348 | 1.1775 | -2.0048 | 1.2482 | 0.108 |
| NT vs MA | -1.8000 | 0.4915 | -2.8015 | 0.3846 | 1.0015 | 0.6241 | 0.109 |
| NT vs VCL | -0.1759 | 0.3587 | -4.9691 | 61.7195 | 4.7931 | 61.7217 | 0.938 |
| EA vs MA | 0.2500 | 0.8189 | 0.1530 | 251.3609 | 0.0969 | 251.3617 | 1.000 |
| 2Hz TEAS vs 100Hz | 1.6300 | 0.3092 | 0.8429 | 0.7297 | 0.7870 | 0.8081 | 0.330 |
| 2Hz TEAS vs MA | -0.0627 | 0.2989 | 0.2188 | 0.3262 | -0.2816 | 0.4401 | 0.522 |
| MA vs WA | -0.0063 | 0.4171 | -0.4028 | 0.6873 | 0.3965 | 0.7940 | 0.618 |

**Table S9 Node-splitting test of LH**

| Side | Direct | | Indirect | | Difference | | P |
| --- | --- | --- | --- | --- | --- | --- | --- |
|  | Coef. | Std. Err. | Coef. | Std. Err. | Coef. | Std. Err. |  |
| CM vs 2Hz TEAS | -1.4200 | 0.2641 | -0.4785 | 0.4955 | -0.9414 | 0.5615 | 0.094 |
| CM vs MA | -0.1703 | 0.4303 | -1.0333 | 0.3436 | 0.8629 | 0.5499 | 0.117 |
| CM vs WA | -0.1629 | 0.4173 | -2.0465 | 1.0705 | 1.8835 | 1.1234 | 0.094 |
| SI vs NT | 0.1980 | 0.2598 | 0.6941 | 0.4180 | -0.4961 | 0.4865 | 0.308 |
| SI vs 2Hz TEAS | -1.2891 | 0.2319 | -2.1921 | 0.7034 | 0.9029 | 0.7313 | 0.217 |
| SI vs 100Hz TEAS | -0.1900 | 0.2328 | 0.4899 | 0.7225 | -0.6799 | 0.7728 | 0.379 |
| SI vs MA | -0.8271 | 0.3047 | -0.9098 | 0.4517 | 0.0827 | 0.5487 | 0.88 |
| NT vs 2Hz TEAS | -1.6538 | 0.3333 | -1.7372 | 0.5291 | 0.0834 | 0.6196 | 0.893 |
| NT vs 100Hz TEAS | -0.3900 | 0.2896 | -1.0965 | 0.9902 | 0.7065 | 1.0580 | 0.504 |
| NT vs MA | -1.3999 | 0.3899 | -1.0466 | 0.3575 | -0.3533 | 0.5290 | 0.504 |
| NT vs VCL | -0.1882 | 0.5321 | -0.9508 | 88.8461 | 0.7626 | 88.8480 | 0.993 |
| EA vs MA | 0.1399 | 0.4130 | -1.3972 | 115.1173 | 1.5372 | 115.1174 | 0.989 |
| 2Hz TEAS vs 100Hz | 1.2400 | 0.2837 | 0.9795 | 0.8683 | 0.2604 | 0.9134 | 0.776 |
| 2Hz TEAS vs MA | 0.1864 | 0.3668 | 0.6724 | 0.2980 | -0.4860 | 0.4815 | 0.313 |
| MA vs WA | -0.0801 | 0.4704 | 1.7097 | 0.8827 | -1.7899 | 0.9972 | 0.073 |

**Table S10 Node-splitting test of T**

| Side | Direct | | Indirect | | Difference | | P |
| --- | --- | --- | --- | --- | --- | --- | --- |
|  | Coef. | Std. Err. | Coef. | Std. Err. | Coef. | Std. Err. |  |
| CM vs 2Hz TEAS | -1.0400 | 1.4223 | -0.5589 | 1.5522 | -0.4810 | 2.1053 | 0.819 |
| CM vs MA | 0.5092 | 1.2445 | 0.1945 | 1.6036 | 0.3147 | 2.0285 | 0.877 |
| CM vs WA | 0.9601 | 1.0225 | 0.0063 | 4.0955 | 0.9538 | 4.2073 | 0.821 |
| SI vs NT | 0.1130 | 1.3365 | -0.4051 | 1.7921 | 0.5182 | 2.2517 | 0.818 |
| SI vs 2Hz TEAS | 0.2396 | 0.8316 | 2.6375 | 1.8702 | -2.3978 | 2.0469 | 0.241 |
| SI vs 100Hz TEAS | 0.5500 | 1.3534 | 0.6332 | 2.9846 | -0.0832 | 3.3225 | 0.980 |
| SI vs MA | 1.7103 | 0.8883 | 2.3202 | 1.7582 | -0.6098 | 1.9703 | 0.757 |
| NT vs 2Hz TEAS | 0.2256 | 1.3792 | 1.5024 | 1.8253 | -1.2767 | 2.3252 | 0.583 |
| NT vs 100Hz TEAS | 0.4600 | 1.2505 | 2.6770 | 4.2399 | -2.2170 | 4.4171 | 0.616 |
| NT vs MA | 2.6000 | 1.7471 | 1.4877 | 1.3527 | 1.1122 | 2.2096 | 0.615 |
| NT vs VCL | 0.7877 | 1.0261 | 2.9244 | 88.0205 | -2.1366 | 88.0275 | 0.981 |
| EA vs MA | -3.6700 | 1.1890 | 0.8216 | 141.0653 | -4.4916 | 141.0690 | 0.975 |
| 2Hz TEAS vs 100Hz | 0.2200 | 1.4092 | -1.1579 | 2.9796 | 1.3779 | 3.4005 | 0.685 |
| 2Hz TEAS vs MA | -0.0733 | 0.9684 | 2.5058 | 0.9871 | -2.5791 | 1.3845 | 0.062 |
| MA vs WA | 0.3782 | 1.2556 | 1.0248 | 2.4219 | -0.6465 | 2.7280 | 0.813 |

1. **Sensitivity analysis after exclusion of varicocele complications**

**Fig. S5 Evidence network diagram for each outcome indicator**

**Table S11 Ranking of SUCRA probabilities for each outcome indicator**

| Intervention | Total effective rate | | Sperm concentration | | Sperm motility a+b% | | Adverse reaction | |
| --- | --- | --- | --- | --- | --- | --- | --- | --- |
|  | SUCRA | RANK | SUCRA | RANK | SUCRA | RANK | SUCRA | RANK |
| WA | **83.4** | **1** | **93.1** | **1** | 62.2 | 5 | **70.0** | **2** |
| EA | **80.3** | **2** | 60.1 | 4 | **78.4** | **1** | 62.4 | 4 |
| 2Hz TEAS | 57.8 | 5 | 48.4 | 7 | **72.9** | **3** | 38.7 | 7 |
| 100Hz TEAS | 48.4 | 6 | 22.8 | 8 | **74.5** | **2** | 39.2 | 6 |
| MB | **72.5** | **3** | **82.9** | **2** | 19.8 | 8 | 20.7 | 10 |
| MA | 32.1 | 8 | 59.4 | 6 | 69.1 | 4 | 28.7 | 9 |
| HBO | 68.1 | 4 | **62.4** | **3** | 60.3 | 6 | 56.8 | 5 |
| CM | 46.2 | 7 | 59.7 | 5 | 43.0 | 7 | 32.4 | 8 |
| SI | 10.2 | 9 | 11.0 | 9 | 13.5 | 9 | **64.2** | **3** |
| NT | 1.0 | 10 | 0.3 | 10 | 6.4 | 10 | **86.8** | **1** |

**Table S12  Network meta-analysis of total effective rate and adverse reaction［RR(95%CI)］**


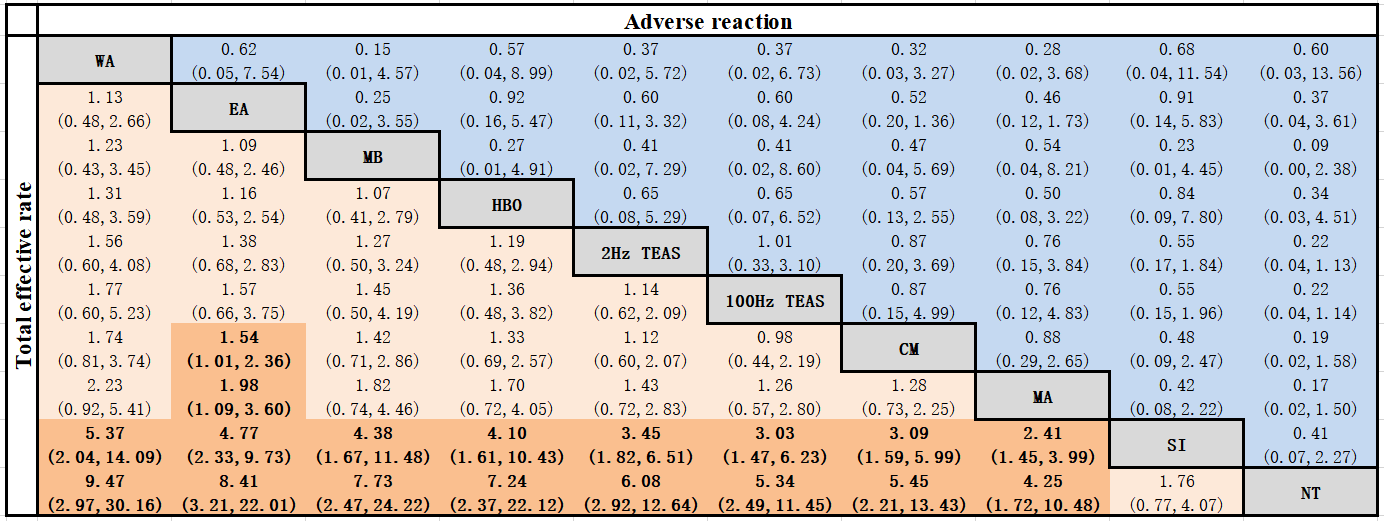


**Table S13  Network meta-analysis of sperm concentration and sperm motility a+b%［MD(95%CI)］**

**
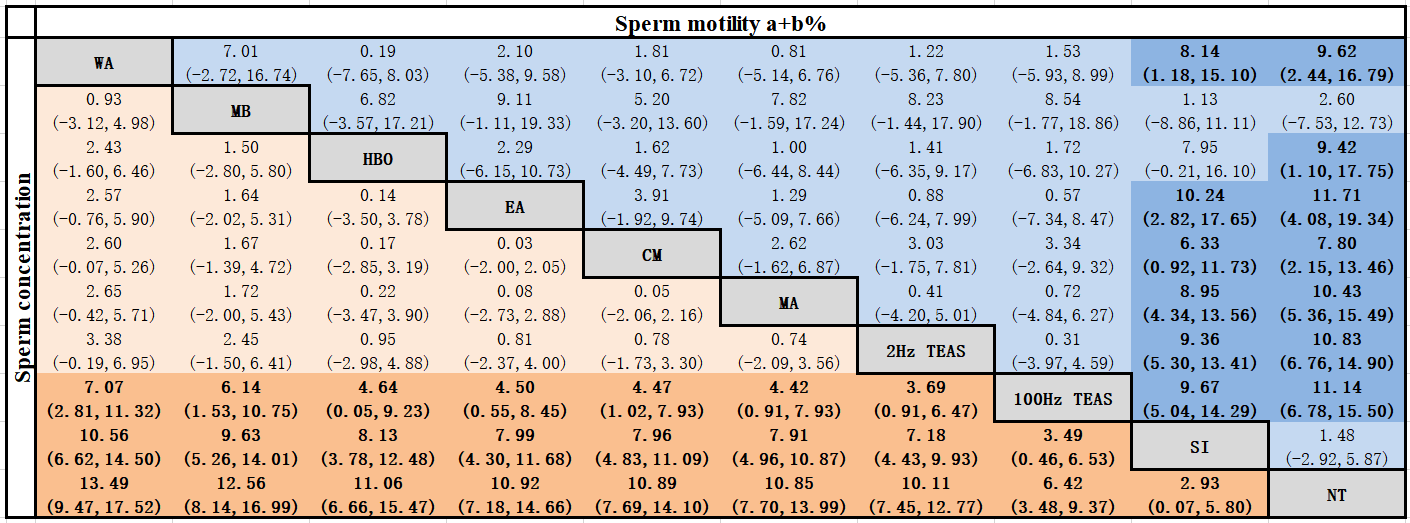
**

**(ii)Sensitivity analysis after exclusion of high-risk literature**

**Fig. S6 Evidence network diagram for each outcome indicator**

**Table S14 Ranking of SUCRA probabilities for each outcome indicator**

| Intervention | Total effective rate | | Sperm concentration | | Sperm motility a+b% | | Adverse reaction | |
| --- | --- | --- | --- | --- | --- | --- | --- | --- |
|  | SUCRA | RANK | SUCRA | RANK | SUCRA | RANK | SUCRA | RANK |
| WA | **91.7** | **1** | **90.8** | **1** | 64.7 | 6 | **68.9** | **2** |
| EA | **84.5** | **2** | **70.1** | **3** | **80.0** | **1** | 64.8 | 4 |
| 2Hz TEAS | 56.7 | 5 | 56.7 | 7 | **72.6** | **3** | 36.3 | 8 |
| 100Hz TEAS | 48.2 | 6 | 30.8 | 8 | **73.3** | **2** | 36.4 | 7 |
| MB | **69.3** | **3** | **80.8** | **2** | 21.6 | 9 | 21.4 | 11 |
| MA | 34.4 | 9 | 64.6 | 5 | 69.9 | 5 | 25.1 | 10 |
| VCL | 42.5 | 8 | 17.0 | 9 | 30.6 | 8 | 55.8 | 6 |
| HBO | 65.8 | 4 | 64.7 | 4 | 72.1 | 4 | 56.2 | 5 |
| CM | 45.9 | 7 | 60.3 | 6 | 47.3 | 7 | 33.2 | 9 |
| SI | 9.9 | 10 | 13.5 | 10 | 13.0 | 10 | **65.3** | **3** |
| NT | 1.1 | 11 | 0.6 | 11 | 5.0 | 11 | **86.5** | **1** |

**Table S15  Network meta-analysis of total effective rate and adverse reaction［RR(95%CI)］**


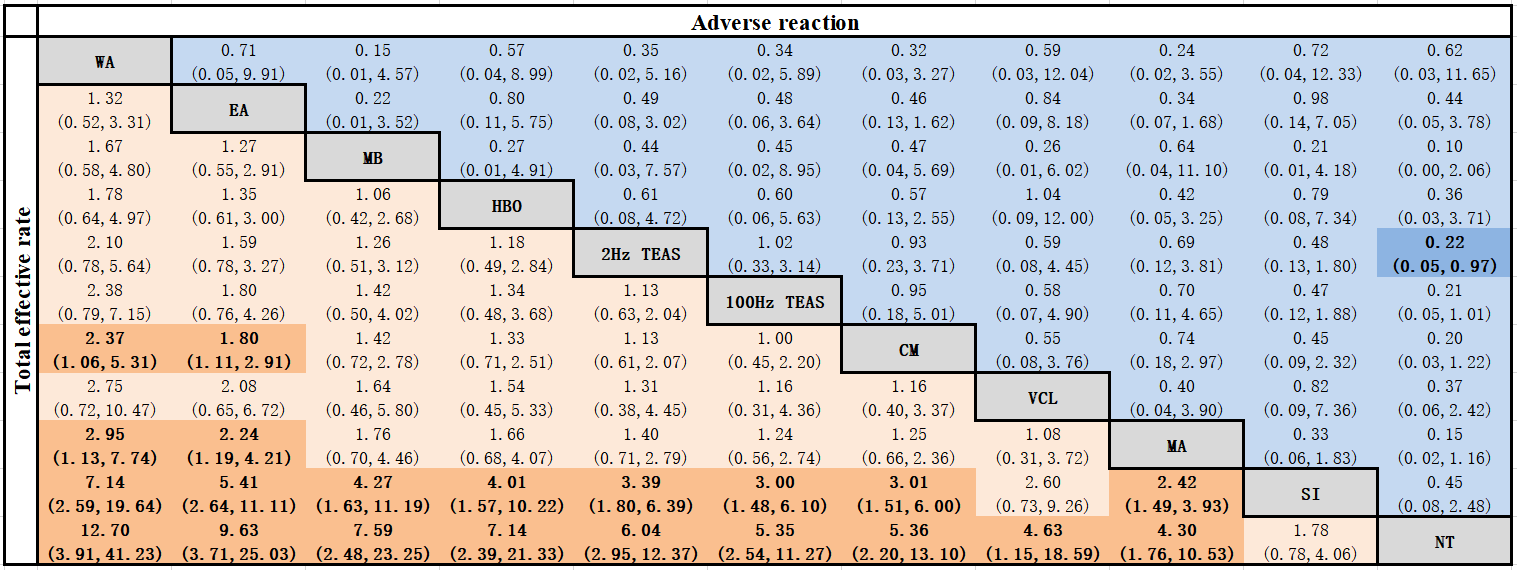


**Table S16  Network meta-analysis of sperm concentration and sperm motility a+b%［OR(95%CI)］**


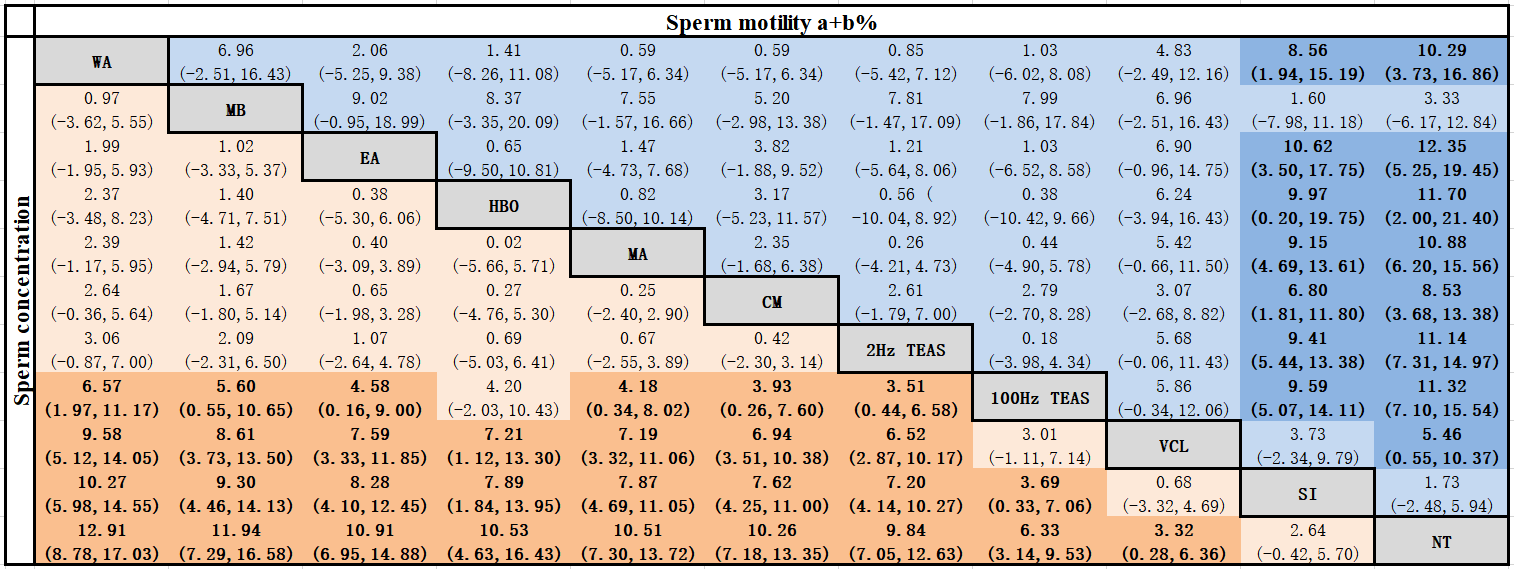


**(ⅲ)Sensitivity analysis after exclusion of small sample sizes literature（<60cases）**


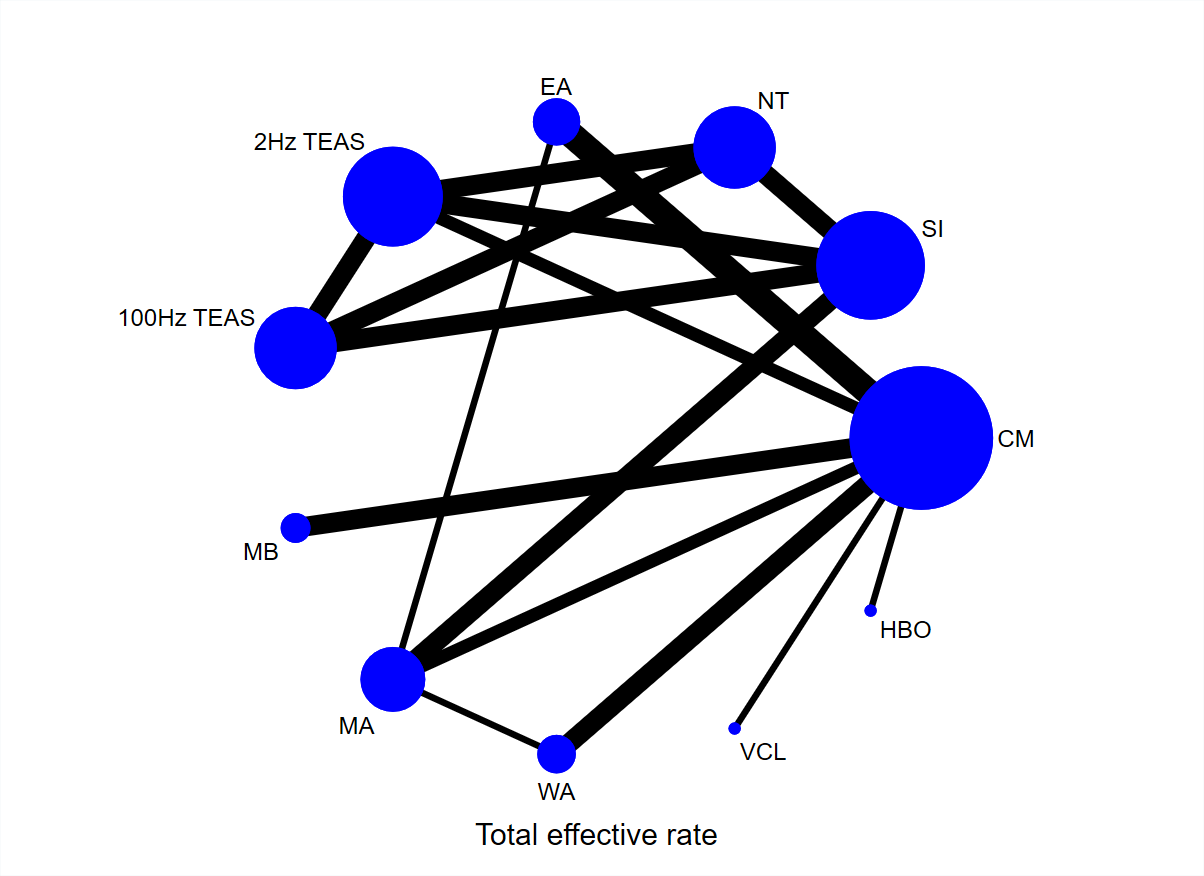

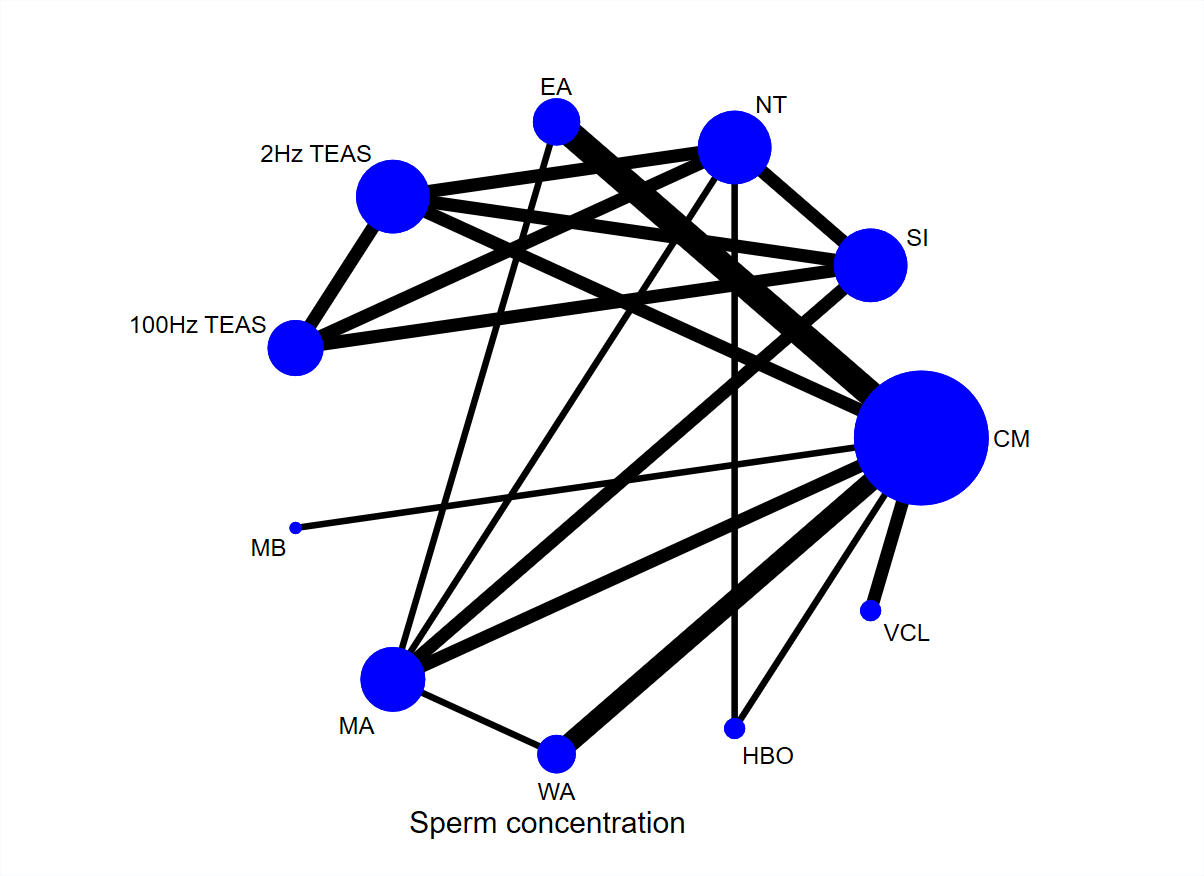


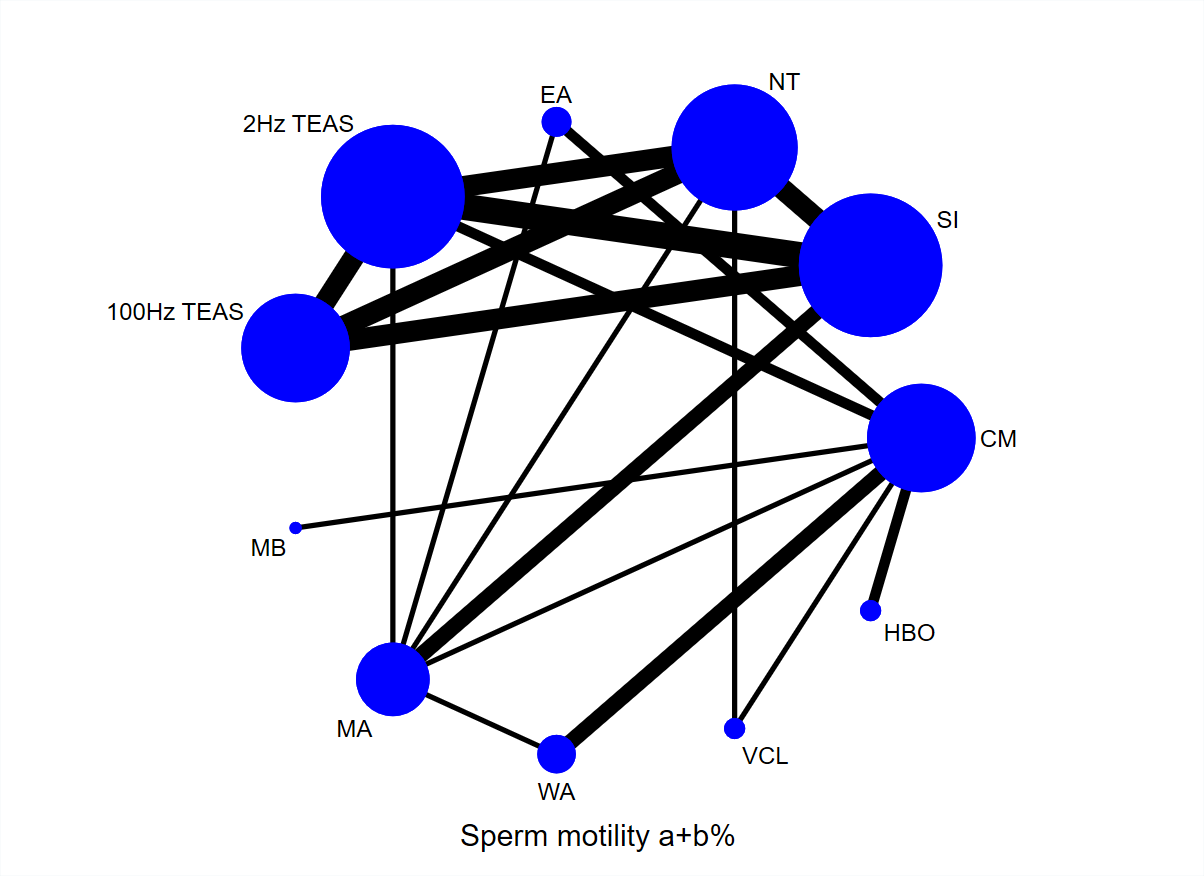

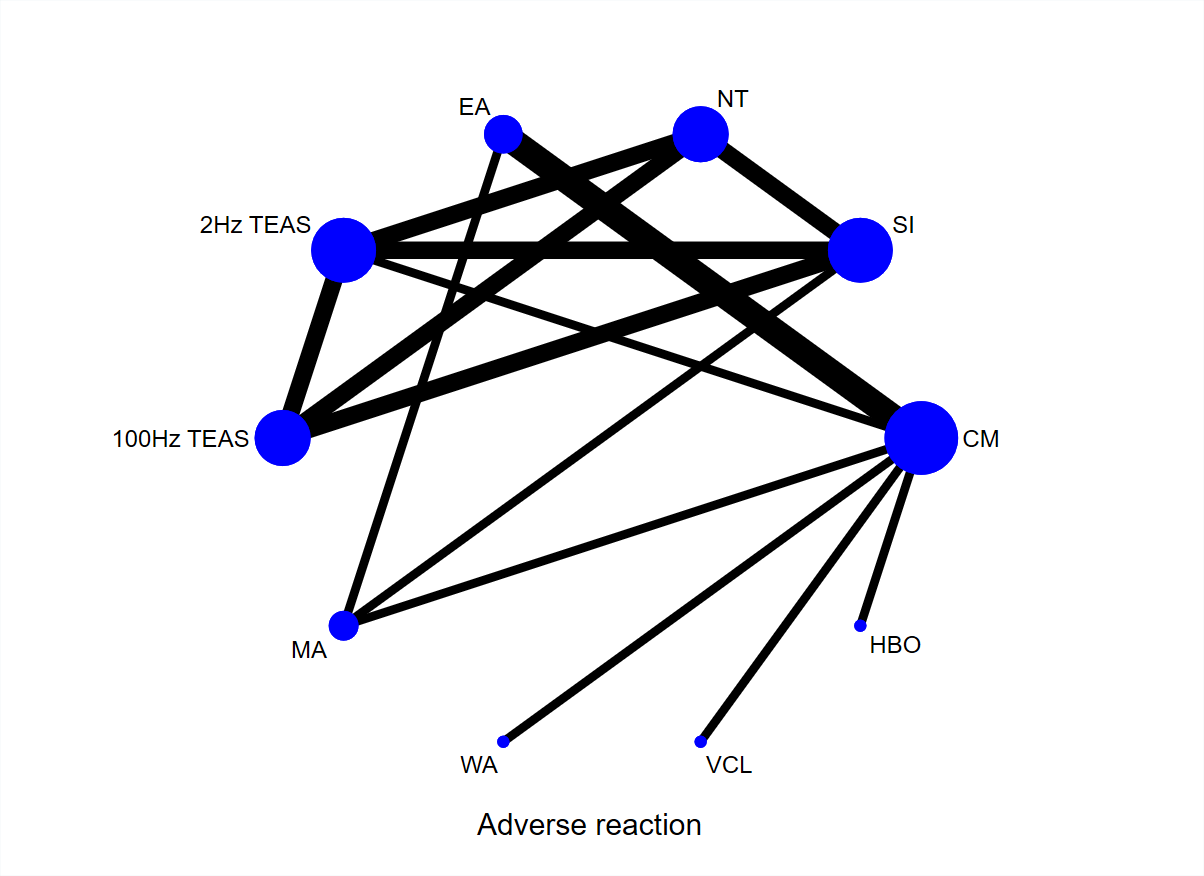


**Fig. S7 Evidence network diagram for each outcome indicator**

**Table S17 Ranking of SUCRA probabilities for each outcome indicator**

| Intervention | Total effective rate | | Sperm concentration | | Sperm motility a+b% | | Adverse reaction | |
| --- | --- | --- | --- | --- | --- | --- | --- | --- |
|  | SUCRA | RANK | SUCRA | RANK | SUCRA | RANK | SUCRA | RANK |
| WA | **82.6** | **1** | **94.8** | **1** | 61.3 | 5 | **65.7** | **2** |
| EA | **79.6** | **2** | **86.1** | **2** | **81.5** | **1** | 57.0 | 4 |
| 2Hz TEAS | 58.5 | 5 | 50.4 | 7 | **75.9** | **3** | 34.8 | 8 |
| 100Hz TEAS | 50.1 | 6 | 24.4 | 8 | **79.7** | **2** | 34.9 | 7 |
| MB | **72.7** | **3** | **70.2** | **3** | 20.2 | 9 | —— | —— |
| MA | 34.4 | 9 | 57.8 | 6 | 71.4 | 4 | 30.1 | 9 |
| VCL | 46.1 | 8 | 23.8 | 9 | 32.4 | 8 | 54.8 | 5 |
| HBO | 66.0 | 4 | 66.7 | 4 | 59.2 | 6 | 51.3 | 6 |
| CM | 49.0 | 7 | 63.8 | 5 | 45.3 | 7 | 25.5 | 10 |
| SI | 10.2 | 10 | 11.9 | 10 | 16.3 | 10 | **61.7** | **3** |
| NT | 0.8 | 11 | 0.0 | 11 | 6.8 | 11 | **84.2** | **1** |

**Table S18  Network meta-analysis of total effective rate and adverse reaction［RR(95%CI)］**


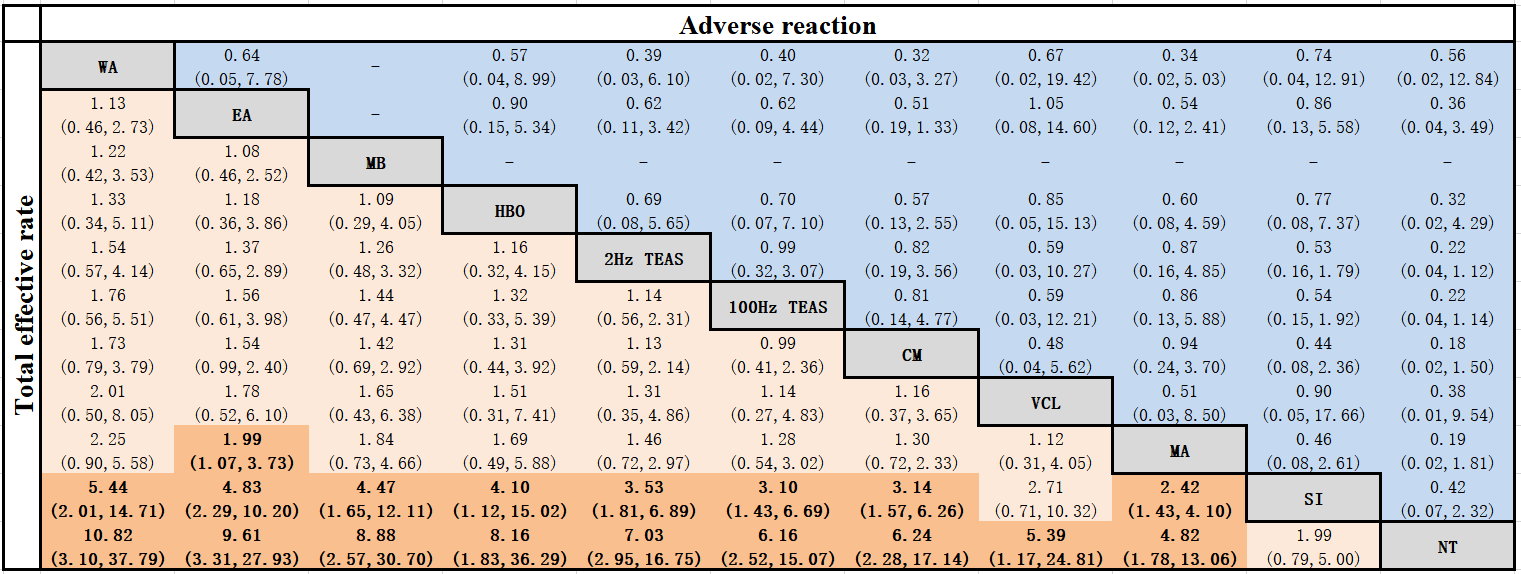


**Table S19  Network meta-analysis of sperm concentration and sperm motility a+b%［OR(95%CI)］**


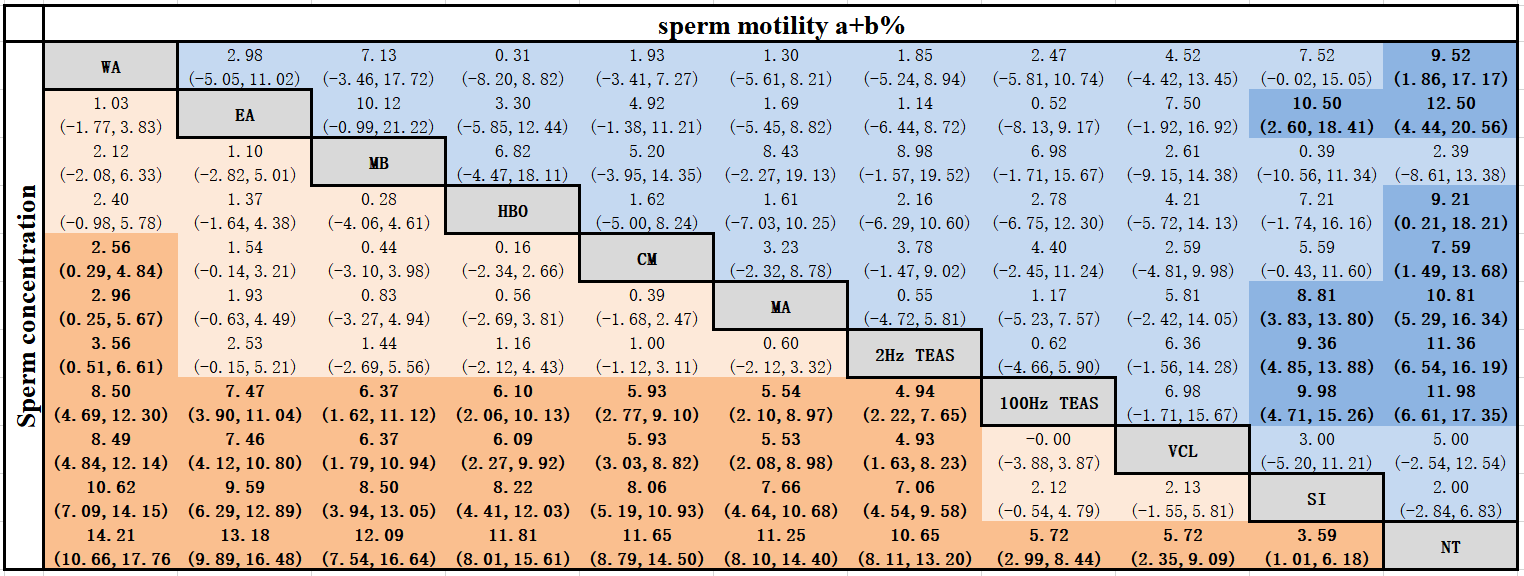


**(ⅳ)Sensitivity analysis after exclusion of short treatment duration literature（≤1month）**

**
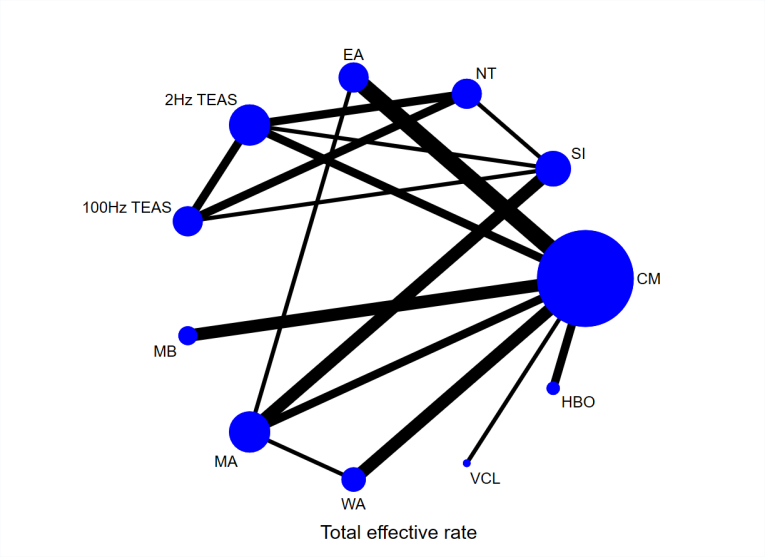

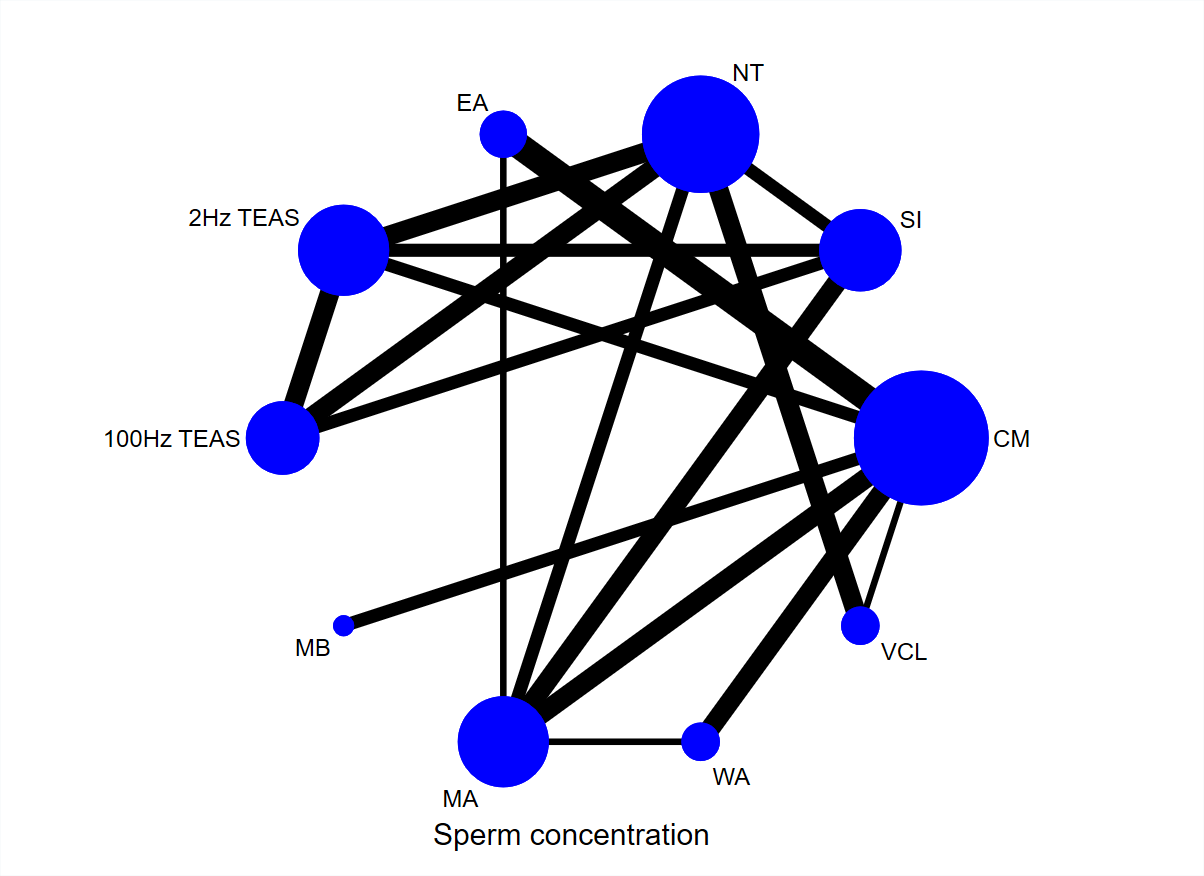
**

**
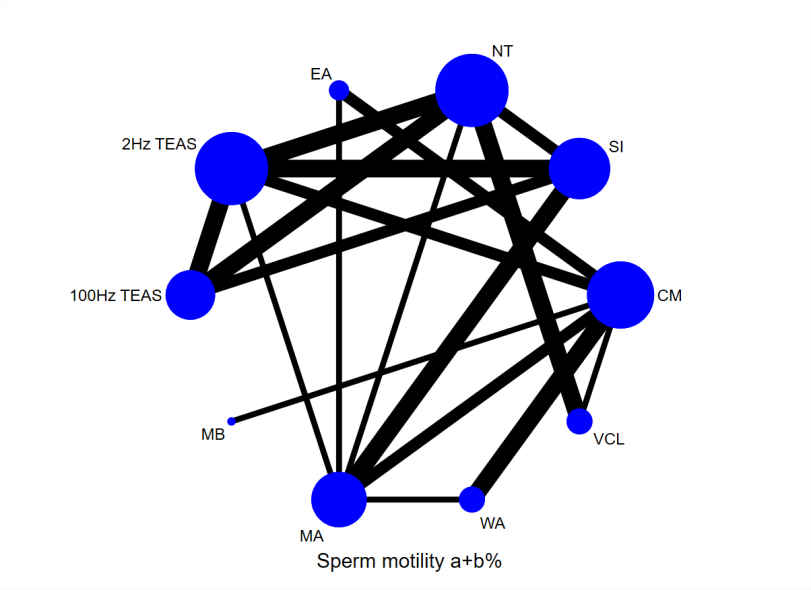

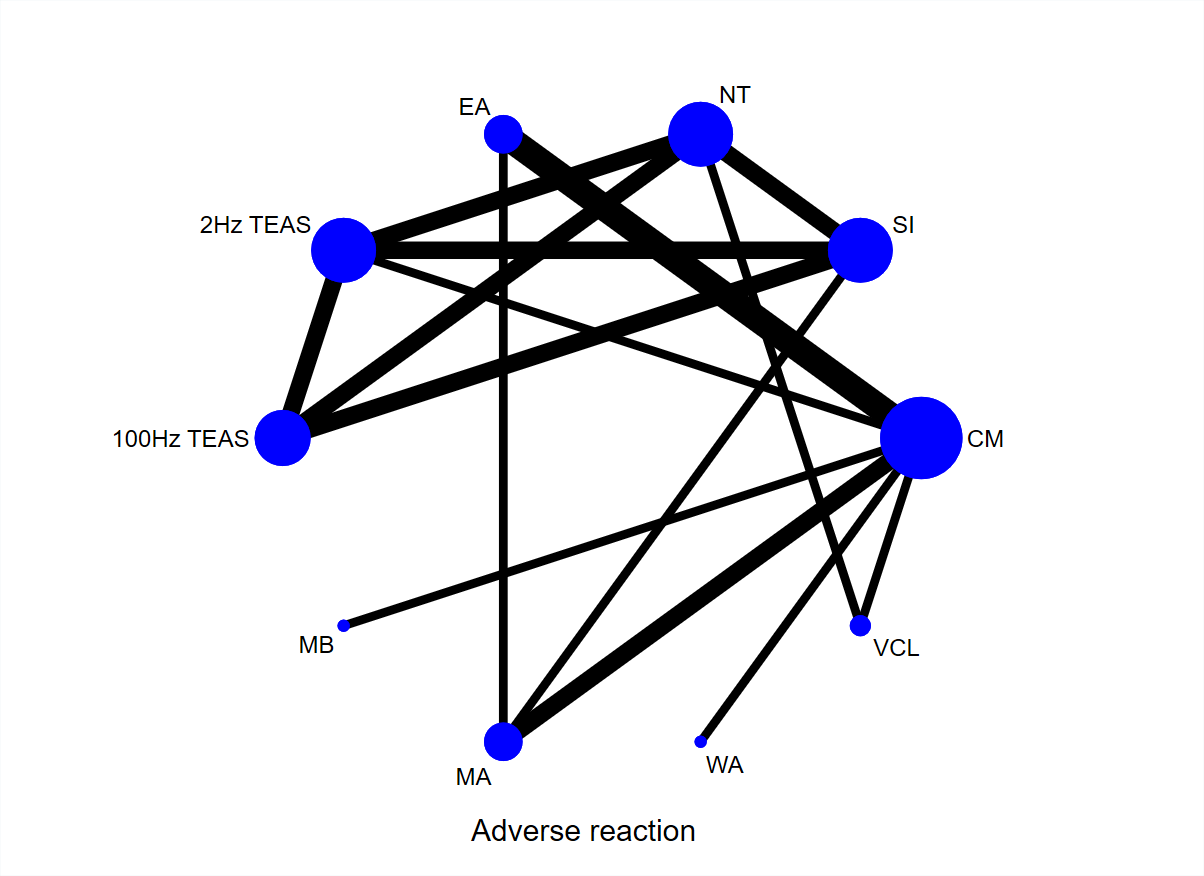
**

**Fig. S8 Evidence network diagram for each outcome indicator**

**Table S20 Ranking of SUCRA probabilities for each outcome indicator**

| Intervention | Total effective rate | | Sperm concentration | | Sperm motility a+b% | | Adverse reaction | |
| --- | --- | --- | --- | --- | --- | --- | --- | --- |
|  | SUCRA | RANK | SUCRA | RANK | SUCRA | RANK | SUCRA | RANK |
| WA | **82.4** | **1** | **93.5** | **1** | 65.2 | 5 | **70.2** | **2** |
| EA | **80.3** | **2** | **65.6** | **3** | **85.1** | **1** | 61.4 | 4 |
| 2Hz TEAS | 52.7 | 6 | 59.0 | 6 | **80.1** | **2** | 38.6 | 7 |
| 100Hz TEAS | 62.1 | 4 | 33.2 | 7 | **75.1** | **3** | 38.7 | 6 |
| MB | **72.1** | **3** | **84.6** | **2** | 24.1 | 8 | 20.4 | 10 |
| MA | 37.5 | 9 | 65.5 | 4 | 71.1 | 4 | 31.9 | 8 |
| VCL | 44.6 | 8 | 18.7 | 8 | 32.2 | 7 | 56.9 | 5 |
| HBO | 57.9 | 5 | —— | —— | —— | —— | —— | —— |
| CM | 48.3 | 7 | 64.4 | 5 | 48.5 | 6 | 28.2 | 9 |
| SI | 10.2 | 10 | 14.9 | 9 | 11.9 | 9 | **64.6** | **3** |
| NT | 1.8 | 11 | 0.6 | 10 | 6.7 | 10 | **89.2** | **1** |

**Table S21  Network meta-analysis of total effective rate and adverse reaction［RR(95%CI)］**


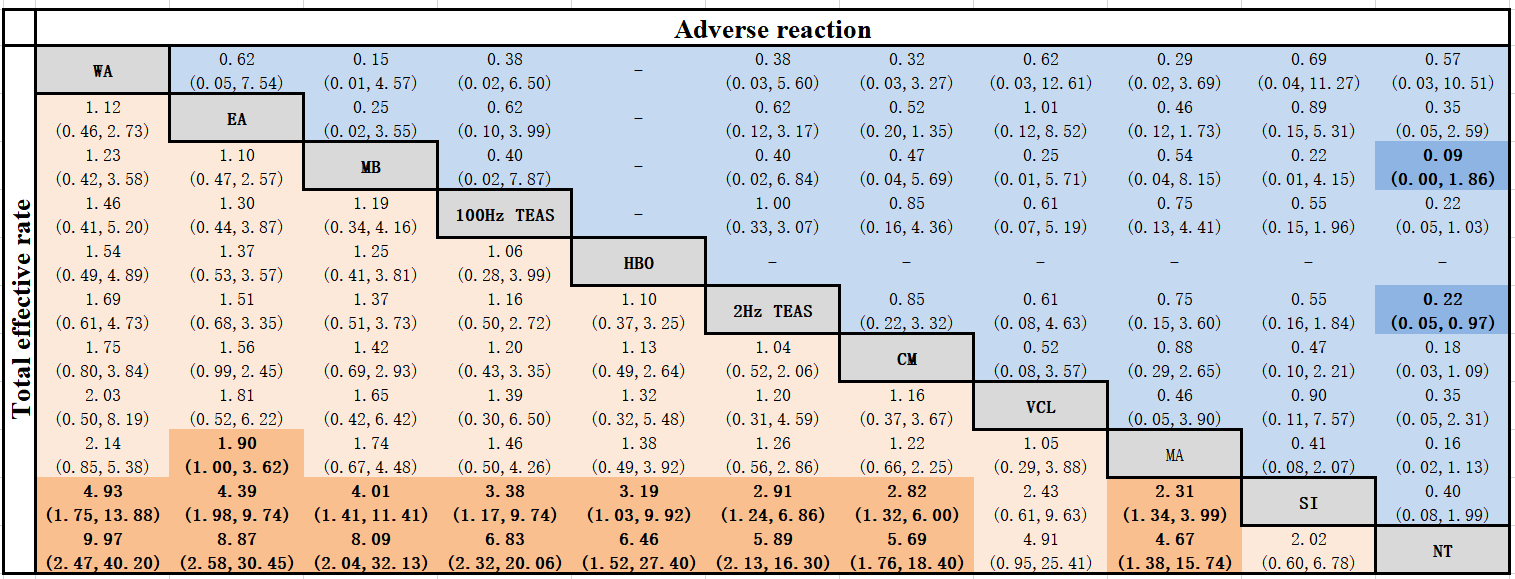


**Table S22  Network meta-analysis of sperm concentration and sperm motility a+b%［OR(95%CI)］**


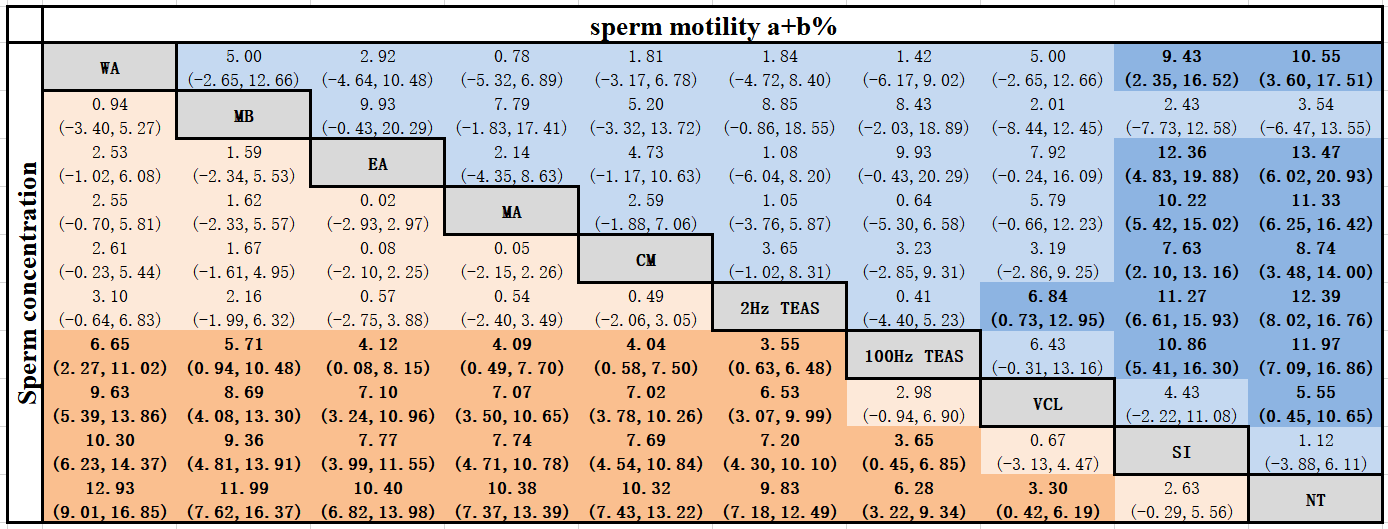


**Fig. S9 GRADE Assessment of Quality of Evidence**


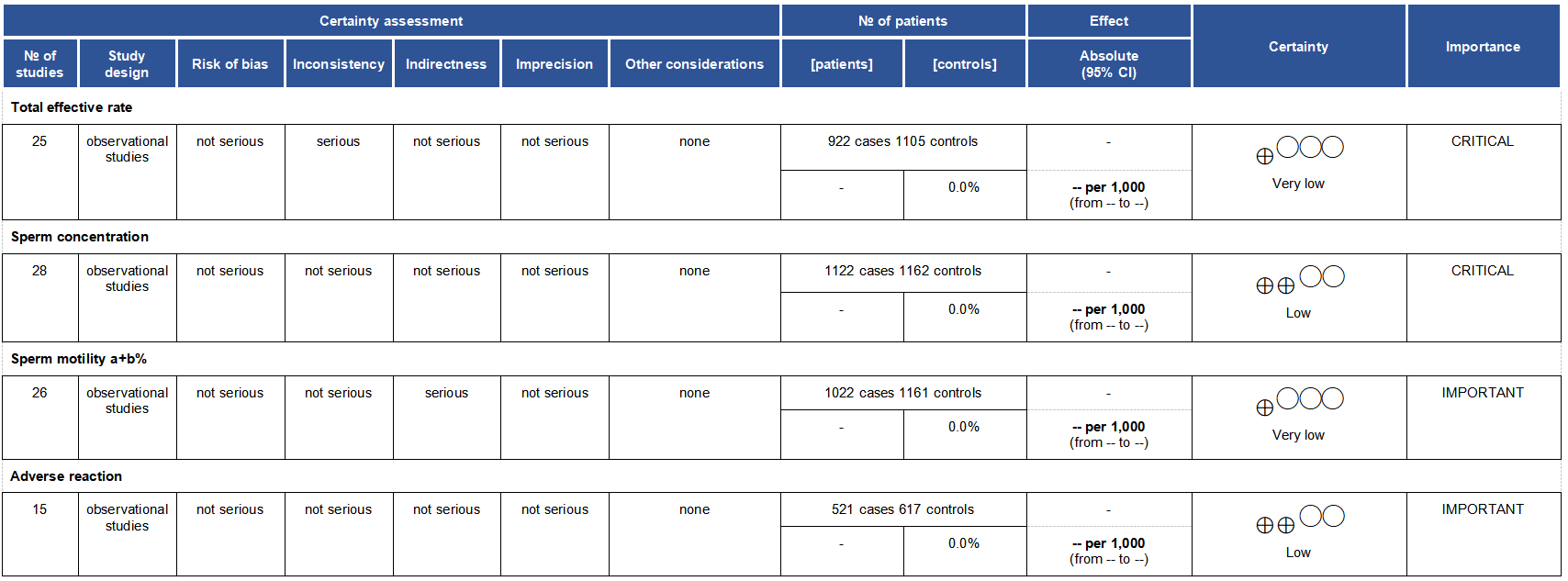


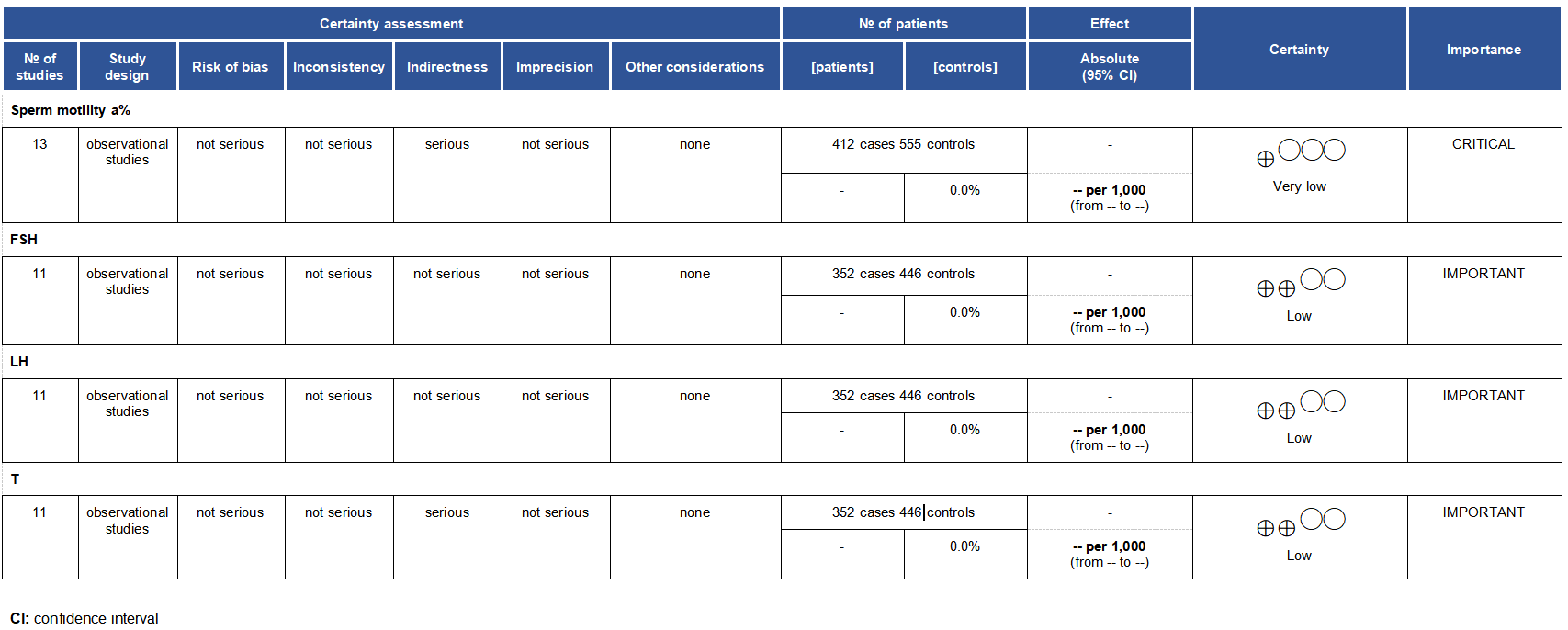


**Table S23 The specific circumstances of the occurrence of adverse reactions**

| **Included Studies** | **Interventions** | **adverse reactions** |
| --- | --- | --- |
| Li C 2020 | EA | Needlesickness 1 case |
| Fang LQ 2021 | 2Hz TEAS | Allergy 2 cases |
| Ding J 2019 | MA | Dizziness 1case; Haematoma 2 cases |
|  | CM | Diarrhoea 1case; Sore throat 2 cases |
| Wang WY 2016 | WA | Needlesickness 1 case |
| Jia YF 2014 | VCL | Scrotal swelling 5 cases |
|  | NT | Scrotal swelling 2 cases |
| Gu YN 2016 | VCL | Haematoma 1 case |
| Chen Y 2021 | HBO | penis ultra-erection 1 case; Diarrhoea 1 case; Barotrauma 1 case |
|  | CM | penis ultra-erection 2 cases; Diarrhoea 2 cases; hypertension 1 case |

**Table S24 Protocol amendment**

| The protocol for this network meta-analysis was registered with PROSPERO (registration number CRD42022314429).After reviewing extensive literature and taking suggestions from reviewers, some of our research methods have been updated and modified based on the protocol.For the sake of transparency, we explained any changes to the protocol, along with supporting reasons. |
| --- |
| 1.Regarding the risk of bias assessment, we adopted the ROB1 assessment method in the protocol.After receiving suggestions from reviewers, we reviewed a large amount of literature and found that ROB2 was used to assess bias in more and more articles.Overall,RoB2 is rich and informative. On the basis of RoB1, the details of each important component of bias assessment are further improved, and the importance of bias assessment in the process of evidence integration and evaluation is emphasized.Therefore, ROB2 was used instead of ROB1 as the bias evaluation method. |
| 2.In the statistical analysis part of the protocol, we used odds ratio (OR) as the effect size of the binary variable.However,More and more articles have adopted RR recently, and some evidence-based medicine experts and reviewers believe that RR is more accurate and precise compared to OR.Therefore,we used RR to analyze the total effective rate and adverse reaction, and compared the results with those previously obtained using OR. We found that there was little difference between the results obtained using OR and RR for these two measures. Taking into account several factors, such as suggestions from reviewers, literature support, and consistency of statistical analysis of articles, RR was used to evaluate two dichotomous variables. |
